# A novel intelligent fault identification method based on random forests for HVDC transmission lines

**DOI:** 10.1371/journal.pone.0230717

**Published:** 2020-03-26

**Authors:** Hao Wu, Qiaomei Wang, Kunjian Yu, Xiaotao Hu, Maoxia Ran

**Affiliations:** 1 Artificial Intelligence Key Laboratory of Sichuan Province, Zigong, China; 2 Automation and Information Engineering, Sichuan University of Science & Engineering, Zigong, China; Wuhan University, CHINA

## Abstract

In order to remedy the current problem of having been buffeted by competing requirements for both protection sensitivity and quick reaction of High Voltage Direct Current (HVDC) transmission lines simultaneously, a new intelligent fault identification method based on Random Forests (RF) for HVDC transmission lines is proposed. S transform is implemented to extract fault current traveling wave of 8 frequencies and calculate the fluctuation index and energy sum ratio, in which the wave index is used to identify internal and external faults, and energy sum ratio is used to identify the positive and negative pole faults occurred on the transmission line. The intelligent fault identification model of RF is established, and the fault characteristic sample set of HVDC transmission lines is constructed by using multi-scale S transform fluctuation index and multi-scale S-transform energy sum ratio. Training and testing have been carried out to identify HVDC transmission line faults. According to theoretical researches and a large number of results of simulation experiments, the proposed intelligent fault identification method based on RF for HVDC transmission lines can effectively solve the problem of protection failure caused by inaccurate identification of traditional traveling wave wavefront or wavefront data loss. It can accurately and quickly realize the identification of internal and external faults and the selection of fault poles under different fault distances and transitional resistances, and has a strong ability to withstand transitional resistance and a strong ability to resist interference.

## 1 Introduction

Because of the vast territory of China, the unbalanced distributions of energy and load center determine the wide application of HVDC transmission technology, so that the rational utilization and optimal allocation of resources can be achieved[1]. HVDC transmission lines are long and have a high probability of failure. The correct identification and diagnosis of faults are critical to the smooth and safe operation of the power system. Thus, it is of great significance to study fast, reliable and accurate fault identification methods for HVDC transmission lines.

For a bipolar HVDC transmission line connected on the same pole, there is an electromagnetic coupling effect between the lines between the two poles. After analyzing the amplitude-frequency characteristics of the physical boundary consisted by smoothing reactor and DC filter on both sides of the HVDC transmission line, it is found that high-frequency signals are blocked by physical boundaries[2], which provides ideas to establish criteria by using high frequency energy. According to the attenuation characteristic of HVDC transmission line boundary elements to high frequency transient signals, references [3, 4, 5] respectively use wavelet energy, polar wave information entropy, and high frequency transient energy to quantitatively describe, analyze and estimate fault characteristics, so as to realize the identification of internal and external faults. These methods can effectively identify internal and external faults, but the threshold setting has no theoretical basis and requires numerous simulation verification. Literatures [6,7] propose to use the single-ended specific frequency signal to construct the fault identification criteria for internal and external faults, but this method is greatly affected by the trigger angle and commutation overlap angle of the control system.

S-transform is applied in some references to extract measurement wave impedance[8] and wave impedance phase[9] to realize internal and external fault identification, but such algorithms have higher requirements on sampling frequency and hardware equipment. In reference [10], the wave impedance is calculated and measured using the transient band component around the tuning point of the DC filter to realize the internal and external fault identification. Although it shows a strong ability to withstand transitional resistance, it fails to discuss the performance of the criteria under the circumstances of data loss and noise interference. In reference[11], with the use of the characteristics of the huge differences in impedance angle at the peak frequency, a vertical protection scheme based on peak frequency is proposed.

References [12,13] realize the identification of internal and external faults by calculating the similarity of the waveforms. However, when data perturbation occurs, the errors of the similarity are large, and misjudgments are liable to occur. In reference[14], the fault identification criterion of the internal and external faults is constructed by using the linear mode voltage reverse traveling wave mutation amount, and the selection of fault poles is realized by the ratio of linear mode and ground mode voltage traveling wave. Since the ground mode wave is susceptible to noise and other factors, the anti-interference ability of this method has yet to be verified. In reference[15], the characteristics of the differences of the reactive energy of the transmission line in the case of internal and external faults are analyzed theoretically. It is proposed that fault identification can be realized because the reactive powers at opposite ends of the transmission line have opposite polarity in the case of internal faults, while the reactive powers have same polarity in the case of external faults. Literature[16] combines the harmonic equivalent circuit of HVDC transmission system and its control strategy with the amplitude-frequency characteristics of typical DC filter, and uses the polarity difference of the characteristic harmonic currents at both ends to realize fault identification. In literature[17], on the basis of the distributed parameter model, differences of differential currents in the case of internal and external faults are used to achieve fault identification, and it turns out to have a certain resistance to transitional resistance.

In literature[18], Utilizing the ability of intelligent algorithms to learn features, a fault identification method for HVDC transmission lines based on multi-resolution singular spectrum entropy and Support Vector Machine (SVM) is proposed to identify internal and external faults, and small sized sample data is applied to identify the faults occurred on the transmission line. Since sample sizes of training and testing used in this method are small, the identification of the internal and external faults and the fault poles selection cannot be performed simultaneously. In reference[19], the standard deviation of the voltage and current signals in the short-term window before and after the fault is used as the input vector of Support Vector Machine (SVM), and the SVM multivariate classifier module is established to determine the fault type. At the same time, the SVM regression algorithm is used to realize fault location. A complete protection scheme for fault detection, classification and ranging of HVDC transmission lines based on SVM is proposed. However, its anti-transitional resistance and anti-interference ability have yet to be verified. In reference [20], the voltage and current signals on the inverter side is used and the K-means data description (KMDD) method is applied to detect and classify internal faults in the bipolar HVDC transmission line. The method can accurately identify the internal fault pole of the transmission line, and the performance in real-time detection is excellent, but the external faults identification is not being considered.

With the rapid development of computer software and hardware technology, intelligent algorithms have made important progress in speech identification, image processing, and condition monitoring, relying on their powerful learning performance. Intelligent algorithms have become research hotspots in various fields, but research on fault identification of HVDC transmission lines are limited. With the concept of "Strong Smart Grid" and "Ubiquitous power Internet of things", it is believed that the intelligent fault identification model has broad application prospects in the future development of smart grids. Therefore, in this paper, a new intelligent fault identification method based on RF for HVDC transmission lines is proposed by analyzing the transmission process of fault traveling waves in the case of internal and external faults. This method uses the multi-scale S-transform fluctuation index feature and energy sum ratio features to reflect the combined feature vector of the DC transmission line, and establishes a random forest intelligent fault identification model. And the fault diagnosis can be performed by inputting the combined feature vectors of the fault samples into the intelligent fault identification model. Theoretical analysis and numerous simulation results show that the proposed new method can accurately and quickly realize internal and external fault identification and fault pole selection under different fault distances and transitional resistances, and has a strong ability to withstand transitional resistance and a strong ability to resist interference.

## 2 Analysis of traveling wave characteristics of HVDC transmission line

### 2.1 Bipolar HVDC transmission system structure

**Fig 1:**
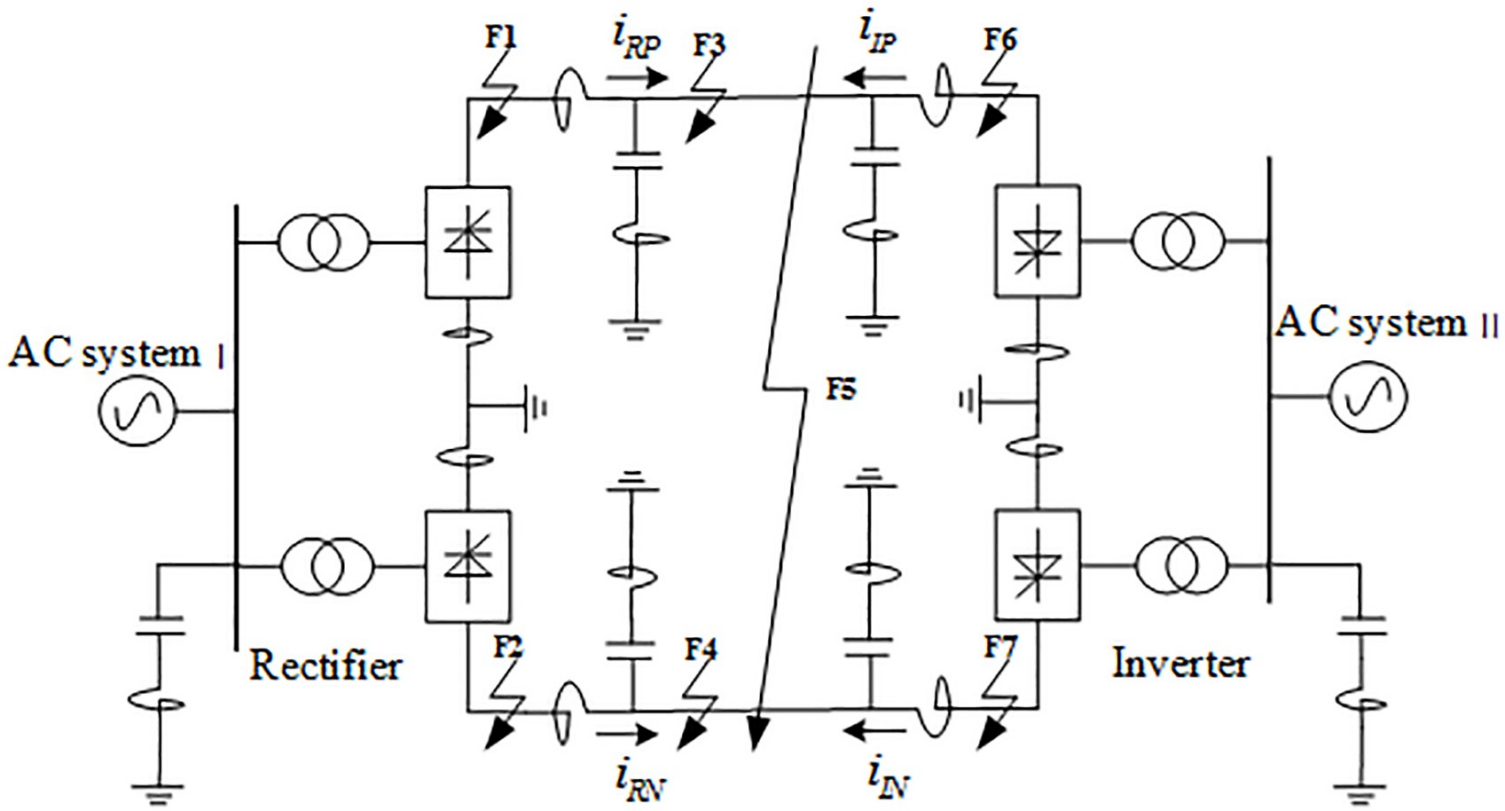
Bipolar HVDC transmission system structure.

The schematic diagram of the bipolar HVDC transmission system is shown in Fig 1. In Fig 1, *i*_*RP*_,*i*_*RN*_ and *i*_*IP*_,*i*_*IN*_ represent the positive and negative polar current on the rectifier side and the inverter side, respectively. *R* and *I* represent the rectification side and the inverter side, respectively, and *P* and *N* represent the positive pole and the negative pole, respectively. *F*_1_~*F*_7_ is the fault points, where *F*_1_~*F*_2_ represent the positive and negative ground faults outside the rectification side, *F*_3_~*F*_4_ represent the positive and negative ground faults in the zone, *F*_5_ represents the short circuit fault between the two poles, and *F*_6_~*F*_7_ represent the positive and negative ground faults outside the inverter side zone. The transmission system failures considered in this paper are mainly unipolar ground faults and short-circuit faults between two poles. The protection device is installed inside the DC line of the converter station, and the internal fault is exemplified by the single-pole ground fault of the DC line and the short-circuit fault between the two poles. The external fault is exemplified by the single-pole ground fault on the outside of the smoothing reactor.

### 2.2 Basic theory of fault traveling wave

When the transmission line fails, the traveling wave starts from the fault point and travels along the line to both sides, and refractions occur where the wave impedance is discontinuous. For any point on the line from the fault point x, we can get the transient voltage and current of this point as [21]:

$$\left\{ \begin{array}{l} u\left(x,t\right)=u_{{}^{+}}\left(x-tv\right)+u_{{}^{-}}\left(x+tv\right)\\ i\left(x,t\right)=i_{{}^{+}}\left(x-tv\right)+i_{{}^{-}}\left(x+tv\right)\\ v=1/\sqrt{LC} \end{array}\right.$$ (1)

In the formula, *t* is the observing time, *L* and *C* are the inductance and capacitance per unit length of the transmission line, respectively, and *u*_+_,*i*_+_ are the voltage and current of the forward traveling wave, and *u*_−_,*i*_−_ are the voltage and current of the backward traveling wave. The high-speed A / D acquisition system is used to collect the fault traveling wave signals, and the S-transform is used to extract the corresponding frequency data in the required data window.

#### 2.2.1 Analysis of traveling wave characteristics when an internal fault occurs

**Fig 2:**
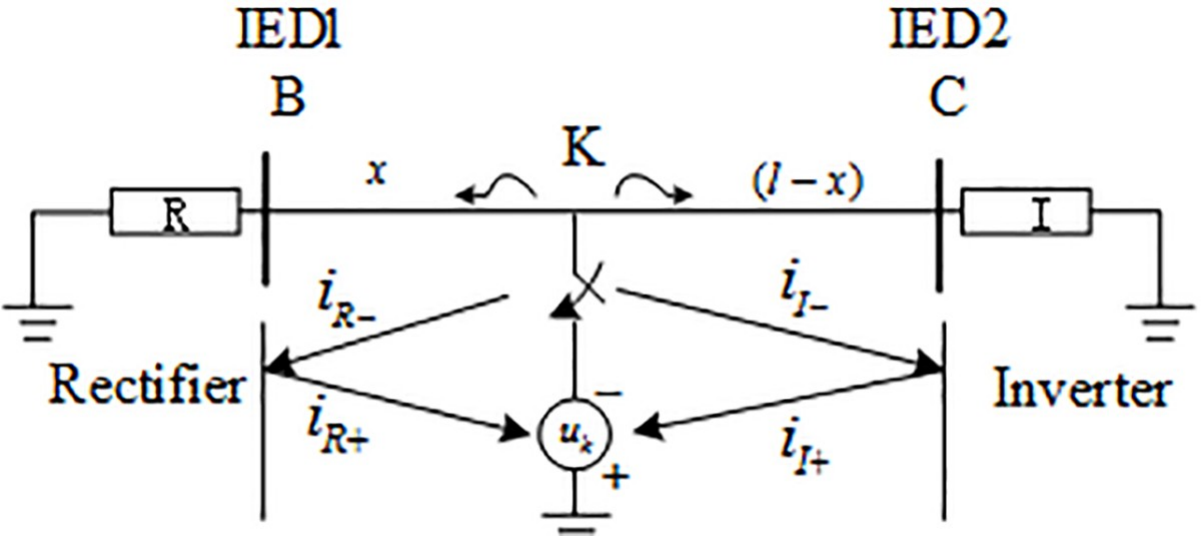
Internal fault additional network.

On the basis of fault superposition theorem, the fault amount measured at the measurement point after the fault can be equivalent to the superposition of the steady-state component before the fault and the additional component of the fault. Due to the presence of an additional voltage source at the fault point, a fault traveling wave propagating from the fault point to the transmission line will be generated. When an internal fault occurs, the fault additional component and traveling wave propagation characteristic are shown in Fig 2, in which IED1 and IED2 respectively represent the protection units installed on the rectifier side and the inverter side of the transmission line, and *u*_*k*_ is the fault superposition voltage source. There are both reverse traveling waves *u*_*R*−_,*i*_*R*−_ and forward waves *u*_*R*+_,*i*_*R*+_ in the fault traveling wave at the IED1 of the rectifier side. Similarly, there are both reverse traveling waves *u*_*I*−_,*i*_*I*−_ and forward traveling waves *u*_*I*+_,*i*_*I*+_ at the IED2 of the inverter side. The fault voltage traveling wave and current traveling wave at the IED1 and IED2 can be expressed as:

$$\left\{ \begin{array}{l} u_{m}=u_{m-}+u_{m+}\\ i_{m}=i_{m-}+i_{m+} \end{array}\right.(m=R,I)$$ (2)

According to the attenuation characteristic of HVDC system transmission lines and boundary elements to fault transient signals[6,7,22], when an internal fault occurs, the fault traveling wave reach IED1 and IED2 by the transmission line attenuation, as a consequence, the attenuation amplitudes of the transient signal measured from IED1 and IED2 are small.

#### 2.2.2 Analysis of traveling wave characteristics when an external fault occurs

**Fig 3:**
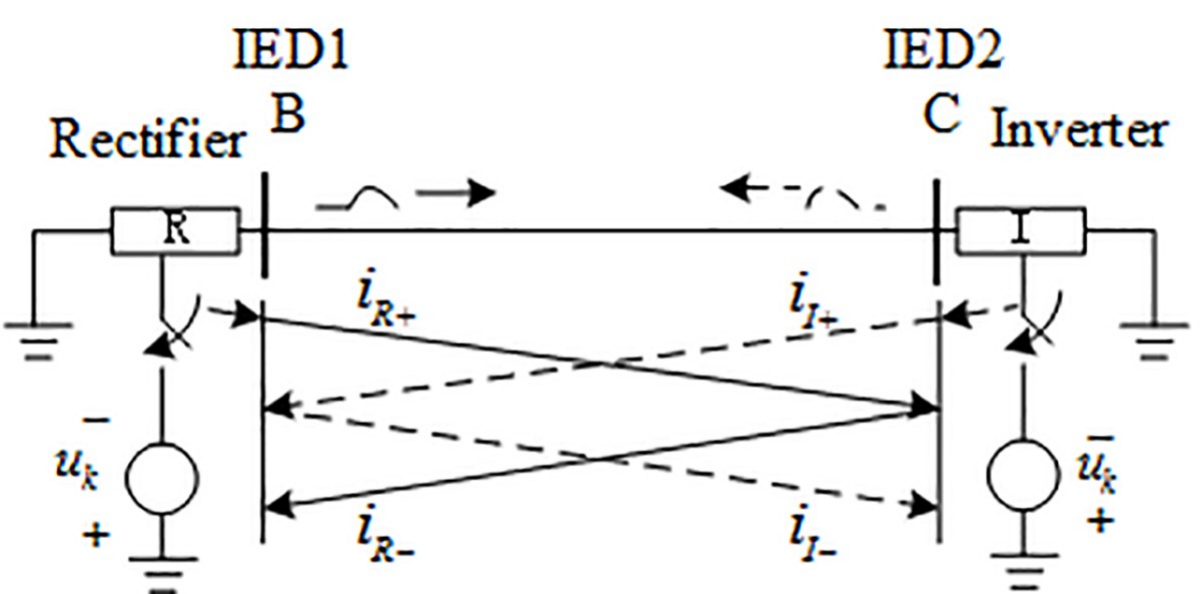
External fault additional network.

The additional components of the fault and the traveling wave propagation characteristics of the HVDC transmission line when an external fault occurs are shown in Fig 3 (the solid line indicates the outside of the rectification side area and the broken line indicates the outside of the inverter side area). According to the attenuation characteristics of the fault signal of the transmission line and its boundary, it should be pointed out that the boundary of the DC transmission line has an obvious attenuation effect on the high-frequency component (10 kHz and above) in the fault transient signal [6,7,22]. When an external fault (take a fault located outside the rectifier for example) occurs, the fault traveling wave reaches IED1 by the attenuation of the boundary element located on the rectifier side, and reaches the IED2 by the double attenuation of the boundary element and the transmission line, so the signal attenuation amplitudes measured at both ends are large.

## 3 Calculation of fluctuation index and energy and ratio based on S-transform

For bipolar DC transmission systems, in order to avoid the effects of electromagnetic coupling, phase-mode conversion techniques are needed to decouple the coupled two-pole lines into separate single-phase systems. In this paper, Karenbauer transform is used to decouple the fault current traveling wave. Taking the rectifier side fault current traveling wave as an example, the decoupling formula of Karenbauer transform can be derived as:

$$\left[\begin{array}{l} i_{G}\\ i_{L} \end{array}\right]=\frac{\sqrt{2}}{2}\left[\begin{array}{l} 1\;1\\ 1\;-1 \end{array}\right]\left[\begin{array}{l} i_{R1}\\ i_{R2} \end{array}\right]$$ (3)

In the formula, *i*_*G*_ and *i*_*L*_ are the ground mode and the linear mode current component, respectively, *i*_*RP*_ and *i*_*RN*_ indicate the positive and negative currents measured at the rectifier side protection installation, and *R* represents the rectifier side, and *P* and *N* indicate the positive and negative poles, respectively. In this paper, the linear mode is selected for discrete S transform, and the fault current traveling waves at multiple characteristic frequencies are selected to calculate the multi-scale S-transform fluctuation index.

### 3.1 Basic principle of S-transform

S transform is a reversible local time-frequency analysis method, which is an extension of the ideas of continuous wavelet transform (CWT) and short-time Fourier transform (STFT) [23]. Assuming the continuous time signal is *h*(*t*), the continuous S transform *S*(*τ*,*f*) of time signal *h*(*t*) is defined as:

$$\{\begin{array}{c} S(\tau ,f)=\int_{-\infty }^{\infty }h(t)g(\tau -t,f)e^{-i2\pi ft}dt\\ g(\tau ‐t,f)=\frac{\left|f\right|}{\sqrt{2\pi }}e^{-\frac{{\left(\tau -t\right)}^{2}}{2\sigma^{2}}} \end{array}$$ (4)

In the equations above, *τ* is a parameter that controls the position of the Gaussian window on the time axis, *f* is a continuous frequency, *t* is time, *i* is an imaginary unit, *σ* = 1/|*f*|, and *g*(*τ*-*t*,*f*) are Gaussian window functions. The height and width of the S-transformed Gaussian window can change with frequency to overcome the shortcomings of fixed height and width of STET window.

Sampling the signal *h*(*t*) can obtain the discrete time series *h*[*kT*](*k* = 0,1,2,⋯,*N*−1). Then the discrete Fourier transform function of *h*[*kT*] is:

$$h[\frac{n}{NT}]=\frac{1}{N}\sum_{k=0}^{N-1}h[kT]e^{-j\frac{2\pi kn}{N}}$$ (5)

And the discrete S-transform of the signal *h*(*t*) can be derived as:

$$\left\{ \begin{array}{l} S[kT,\frac{n}{NT}]=\sum_{r=0}^{N-1}H(\frac{r+n}{NT})e^{-\frac{2\pi^{2}r^{2}}{n^{2}}}e^{{}^{j\frac{2\pi rk}{N}}},n\neq 0\\ S[kT,0]=\frac{1}{N}\sum_{r=0}^{N-1}h(\frac{r}{NT}),n=0 \end{array}\right.$$ (6)

In the equations above, *f*→*n*/*NT*, *τ*→*kT*, *T* is the sampling interval, and *k*,*r*,*n* = 0,1,⋯*N*−1. Signal *h*(*t*) is transformed by S to obtain a complex time-frequency matrix, which reflects the time and frequency domain characteristics of the signal, as well as the amplitude and phase information of the traveling wave in the time domain.

### 3.2 Multi-scale S-transform fluctuation index

The fluctuation index is mathematically defined as the average of the sum of the differences between adjacent signals, and is an indicator used to measure the strength of signal changes. Therefore, the fluctuation index is selected to reflect the variation intensity of fault current traveling wave in this paper. The specific method is to perform Karenbauer transform on the fault current traveling wave, and take the linear mode *i*_*L*_(*t*) to perform discrete S- transform. Since the boundary of the DC transmission line has an obvious attenuation effect on the high-frequency components (10 kHz and above) in the fault transient signal [6,7,22], the sampling data in the short-time window of the fault traveling wave under 8 different characteristic frequencies *f*_*l*_(*l* = 10,11,12,13,14,15,16,17)kHz are extracted on the rectifier side and the inverter side respectively in this paper. The fluctuation index of the corresponding characteristic current traveling wave is calculated, and the fluctuation index is defined as:

$$F_{l}=\sum_{j=1}^{M-1}\left|a_{l}(j+1)-a_{l}(j)\right|$$ (7)

In the formula, *M* represents the number of sampling points in the sampled data window, *l* represents the *l* kHz component of the traveling wave with the implementation of S- transform.

Using the fluctuation index of the above 8 frequencies to form the internal and external fault identification eigenvector, it can be expressed as *F* = (*F*_*R*11_⋯*F*_*R*17_ *F*_*I*11_⋯*F*_*I*17_)_1×16_, in which *R* means rectifier side, *I* represents inverter side, and the eigenvector is defined as multi-scale S-transform fluctuation index vector of the signal *i*_*L*_(*t*).

#### 3.2.1 Analysis of multi-scale fluctuation index when an internal fault occurs

**Fig 4:**
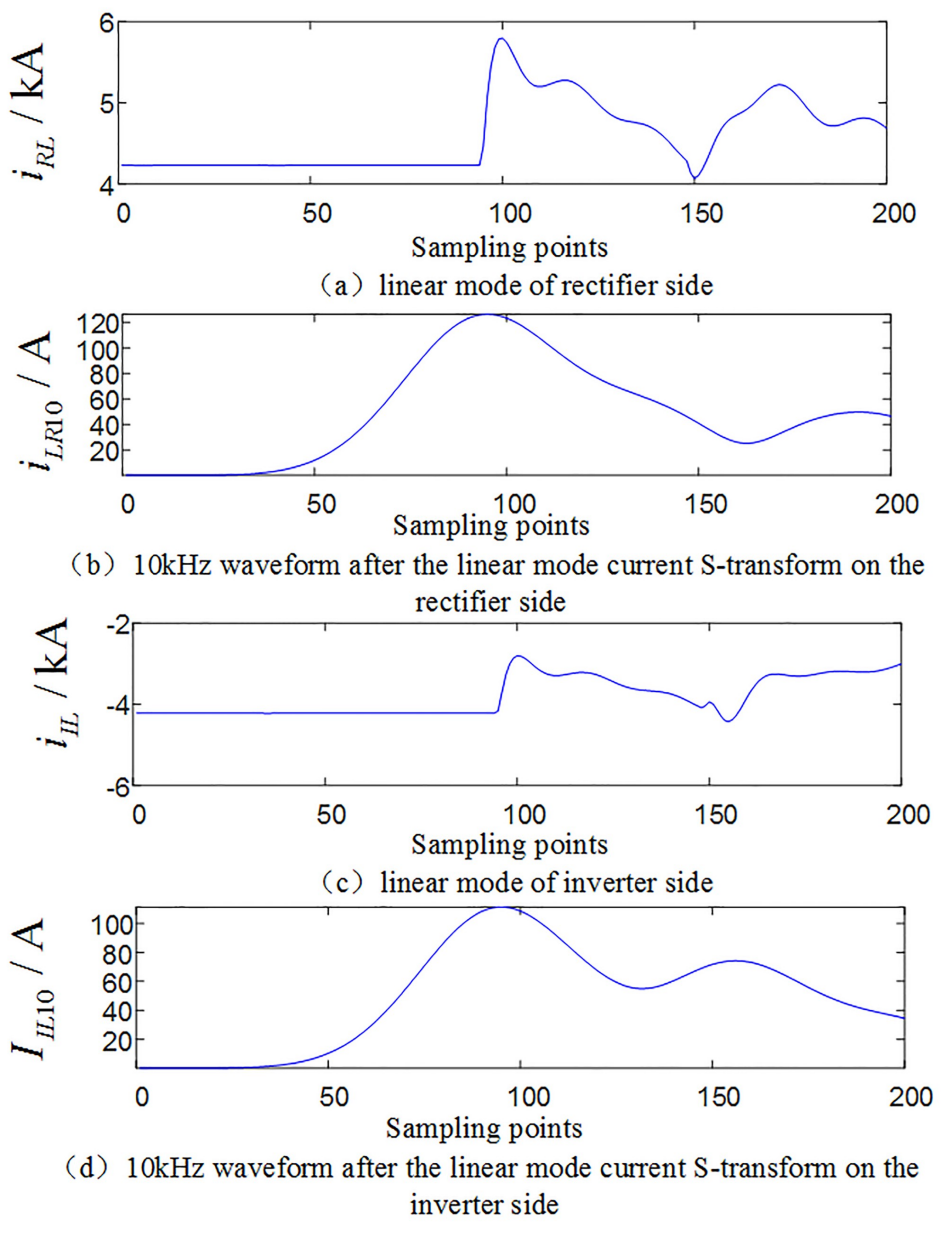
Related current waveforms during an internal fault occurs.

**Fig 5:**
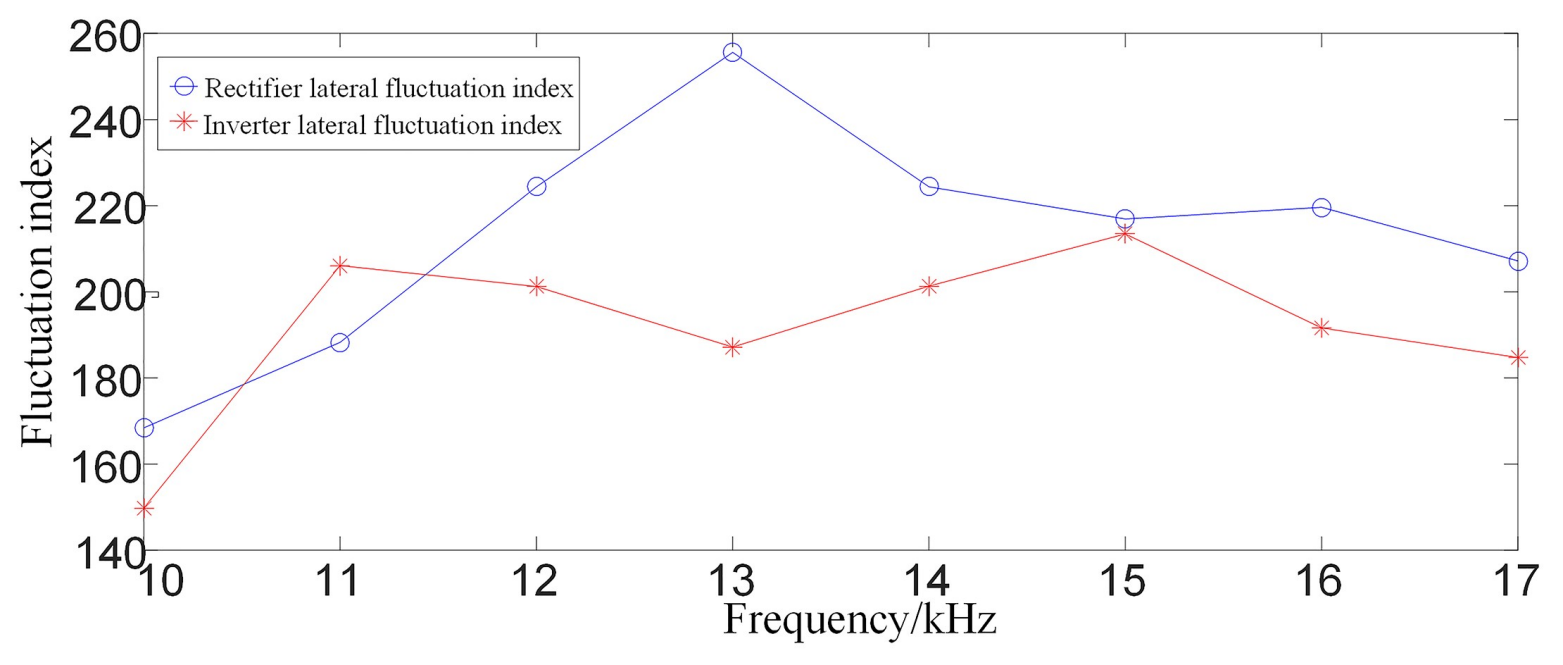
Multi-scale S transform fluctuation index for internal faults.

When an internal grounding fault occurs on positive line F3 (where the transitional resistance is 10Ω, F3 is 200km away from rectifier side protection installation) within the system as shown in Fig 1, the waveforms of the linear mode current and its S-transformed single frequency (in the case of 10 kHz) on the rectifier side and inverter side are shown in Fig 4, and the multi-scale S transform fluctuation index is shown in Fig 5.

#### 3.2.2 Analysis of multi-scale fluctuation index when an external fault occurs

**Fig 6:**
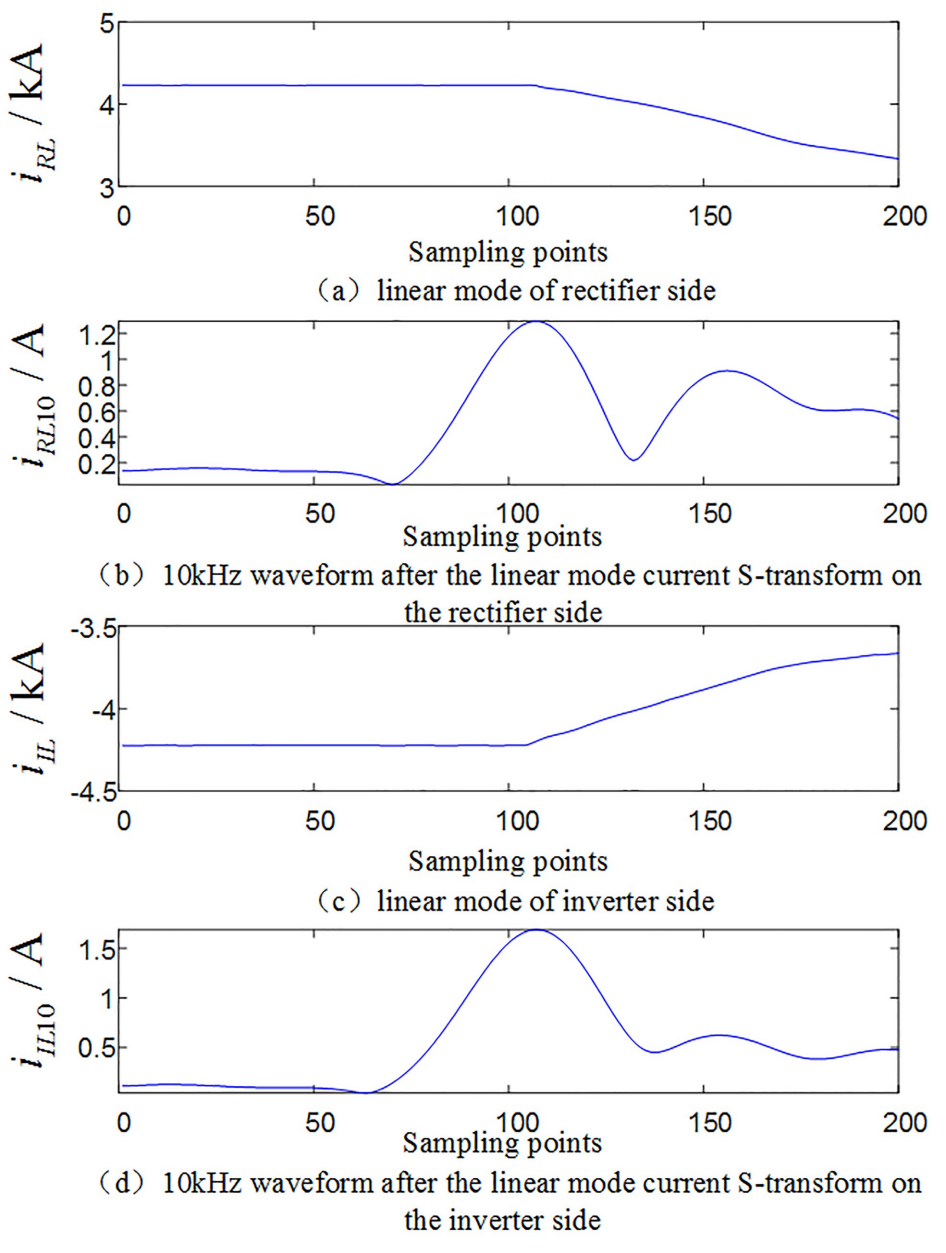
Related current waveforms during an external fault occurs.

**Fig 7:**
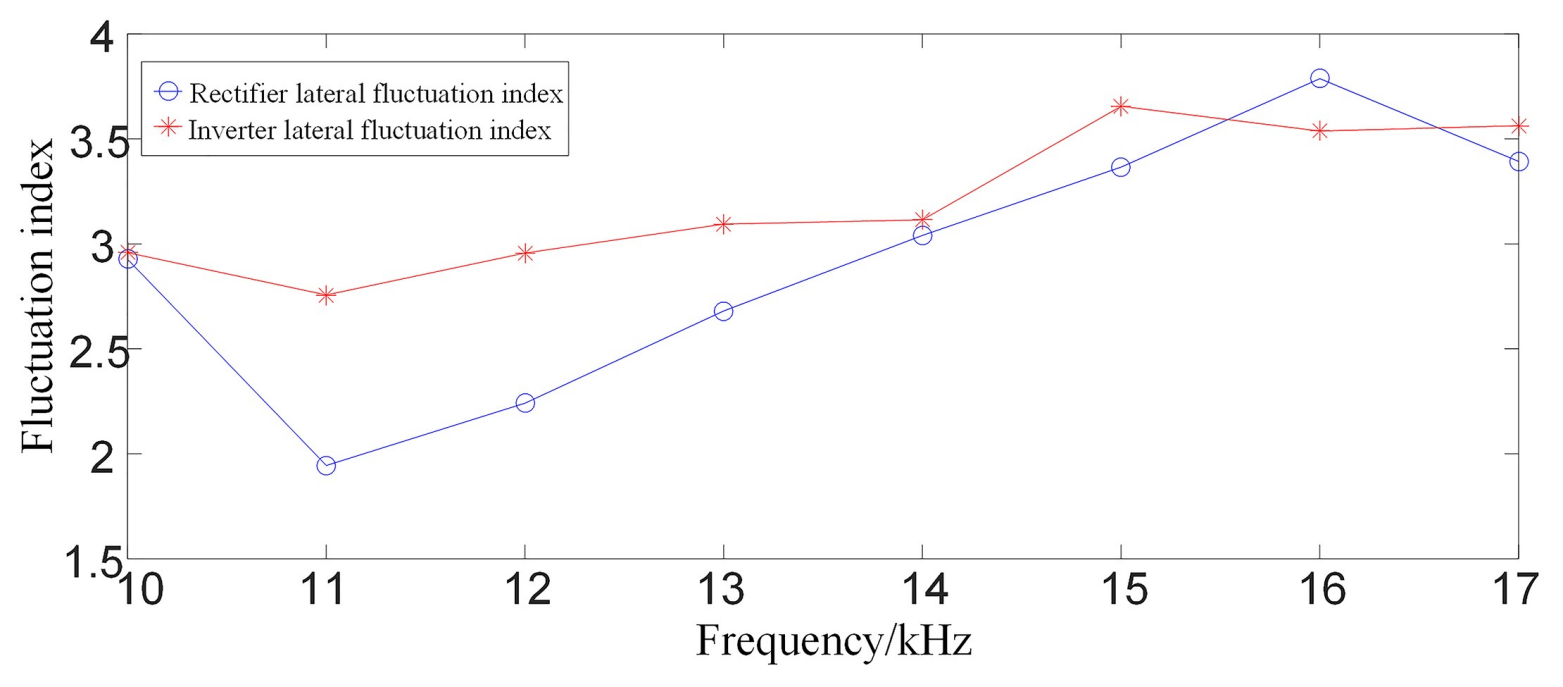
Multi-scale S transform fluctuation index for external faults.

When a ground fault occurs at F1(where the transitional resistance is 10Ω) outside the rectifier side smoothing reactor of the system as shown in Fig 1, waveforms of line mode currents and its S transform characteristic frequency (in the case of 10 kHz) on both sides are shown in Fig 6. Comparing Fig 4(B) with Fig 6(B), Fig 4(D) with Fig 6(D), it can be seen that the amplitude of the characteristic frequency of 10 kHz signal of internal fault is much larger than the characteristic frequency of 10 kHz signal of external fault. And the multi-scale S transform fluctuation index in the case of external fault is shown in Fig 7. Analysis shows that the fluctuation index value of the external fault has a small change range and its value is small. Comparing Fig 5 with Fig 7, the multi-scale fluctuation index of internal fault is much larger than the multi-scale fluctuation index of external fault.

### 3.3 Multi-scale S-transform energy sum ratio

In the bipolar HVDC transmission system, due to the electromagnetic coupling effect between the bipolar lines, when a pole fails, the non-fault pole generates a large number of electromagnetic transients as well. In order to prevent malfunction of the non-fault pole protection device, it is necessary to accurately identify the fault pole. Reference [22] points out that the coupling between bipolar HVDC transmission lines is related to frequency. When the frequency is in the range of [0,100] kHz, the coupling coefficient *ω*_*c*_ increases at first and then decreases, but 0.5<*ω*_*c*_<1 can be met in general. That is, the transient signal detected on the non-fault pole line is always weaker than that of the fault pole line, and the lower the frequency is, the more obvious the difference is. Therefore, this paper uses the multi-scale S-transform energy sum ratio of the two-pole line fault current traveling wave to characterize the fault pole characteristics, and selects the signal of *f*_*j*_(*j* = 0.1,0.2,0.3,0.4,0.5,0.6,0.7,0.8)kHz to calculate the energy sum ratio. The energy and ratio formula is shown in Eq (8):

$$k_{j}=\frac{\sum_{a=1}^{N_{K}}I_{{}_{mPj}}^{2}}{\sum_{a=1}^{N_{K}}I_{{}_{mNj}}^{2}}(m=R,I)$$ (8)

In the equation, *I*_*mPj*_ and *I*_*mNj*_ represent the j kHz components of the positive and negative transmission line current fault traveling wave with the implementation of S transform, respectively, R and I represent the rectification side and the inverter side, respectively, and P and N represent the positive and negative poles, respectively. a = 1 indicates the first sampling point in the 2ms data window taken; *N*_*K*_ is the number of sampling points in the 2 ms data window.

Implement discrete S transform on the positive and negative currents on the rectifier side and inverter side, and the sampling data in the short-time window of the fault traveling wave at 8 different frequencies *f*_*j*_(*j* = 0.1,0.2,0.3,0.4,0.5,0.6,0.7,0.8)kHz are respectively extracted. And then the fault pole identification eigenvector *K* = (*K*_*R*0.1_⋯*K*_*R*0.8_ *K*_*I*0.1_⋯*K*_*I*0.8_)_1×16_ is constructed by using the energy sum ratio at the corresponding frequency of the positive and negative current fault traveling wave, where R represents the rectifier side, I represents the inverter side, and the vector is defined as a multi-scale S-transform energy sum ratio vector.

## 4 Random forests

Due to the limitations and poor stability of the single classifier, the accuracy of the classifier is difficult to increase when it meets certain conditions. Random Forests, with good generalization ability and accuracy, is a combined separator algorithm proposed by Leo Breiman in 2001[24]. It combines Breiman’s "Boot-strap aggregating" idea with Ho's "random subspace" method to improve prediction accuracy by integrating multiple DT models. Using the decision results of multiple DT models, the output results are finally determined by a comprehensive vote of all decision trees.

The random decision tree is the smallest decision-making unit that constitutes a random forest. Its generation can be summarized as two random features: random training samples of each decision tree are generated by Bagging method [25], and the features of training samples are randomly selected to split the nodes of the random decision tree. The construction steps of random forests are as follows:

(1) Use the Booststrap method to resample to generate a training set for each decision tree. Let W be a set containing n different samples {*c*_1_,*c*_2_,⋯,*c*_*n*_}. If a single sample *c*_*i*_ is returned from the set W with a single replacement, and a total of n times are taken to form a new set W*, the probability that the new set does not include a sample *c*_*r*_(*r* = 1,2,⋯,n) is:

$$p={\left(1-\frac{1}{n}\right)}^{n}$$ (9)

$$\underset{n\to \infty }{\lim}p=\underset{n\to \infty }{\lim}{\left(1-\frac{1}{n}\right)}^{n}=e^{-1}\approx 0.368$$ (10)

Therefore, 36.8% of the samples will not be extracted. This will render the decision tree in the random forests not produce a local optimal solution, and the abnormal data will be effectively prevented from appearing in the sample set.

**Fig 8:**
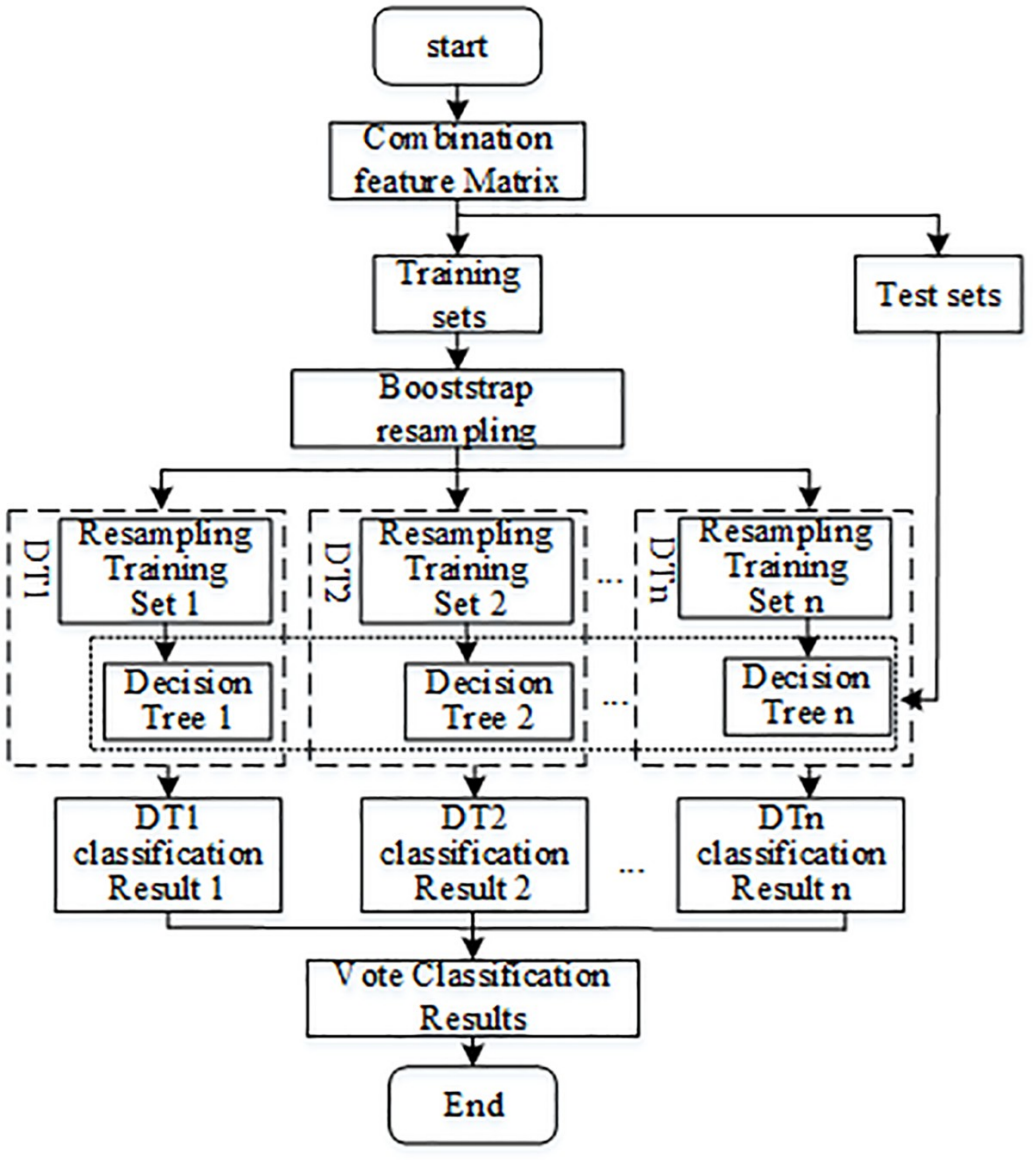
Random forests structure.

(2) Build each decision tree. Before selecting attributes on each non-leaf node, randomly extract q(q<Q) attributes from the Q attributes as the classification attribute set of the current node, and split the node in the best split mode among the q attributes. A complete decision tree is constructed by node splitting, and each decision tree is allowed to grow without pruning until it reaches the leaf node. Using each training set, a corresponding decision tree is generated.

(3) Forming a random forest. Use the constructed decision tree to test the samples, summarize the output category of each decision tree. The most output categories of all decision trees are used as the test sample category. The random forest structure is shown in Fig 8.

In order to realize the internal and external fault identification and fault pole judgment in the HVDC transmission line with the same network, on the basis of multi-simulation verification, this paper selects the optimal decision tree number as 20, and uses the combined feature vector that can reflect both the internal and external fault feature and the fault pole feature as the input vector of the random forest. Among them, the multi-scale S-transform fluctuation index vector is used to reflect the internal and external fault characteristics, and the multi-scale S-transform energy sum ratio vector is used to reflect the fault pole characteristics, so that the same network can be used to solve the fault identification and polarity selection problems. The classification and label numbers are shown in Table 1.

**Table 1:** Random forests classification label.

Features	Internal and external fault identification and poles selection				
Output	external positive pole ground fault (EPG,E = R,I)	external negative pole ground fault (ENG,E = R,I)	internal positive pole ground fault (LPG)	internal negative pole ground fault (LNG)	internal positive and negative poles fault(LPN)
Label	1	2	3	4	5

*E* represents the outside of the line area, *L* represents the line area, *R*, *I* represent the rectification side and the inverter side, *P*, *N* represent the positive and negative poles, and *G* represents the ground fault. For *n* independent classifications, set the number of outputs as *n*, and the corresponding classification labels are 1,2,⋯,n successively, and each label corresponds to one output classification. The fault types studied in this paper can be divided into 5 categories, namely: external positive pole ground fault, external negative pole ground fault, internal positive pole ground fault, internal negative pole ground fault, and the internal fault between the positive and negative poles. Set the output types as 5 and the labels are 1~5.

## 5 Intelligent fault identification algorithm for HVDC transmission lines

**Fig 9:**
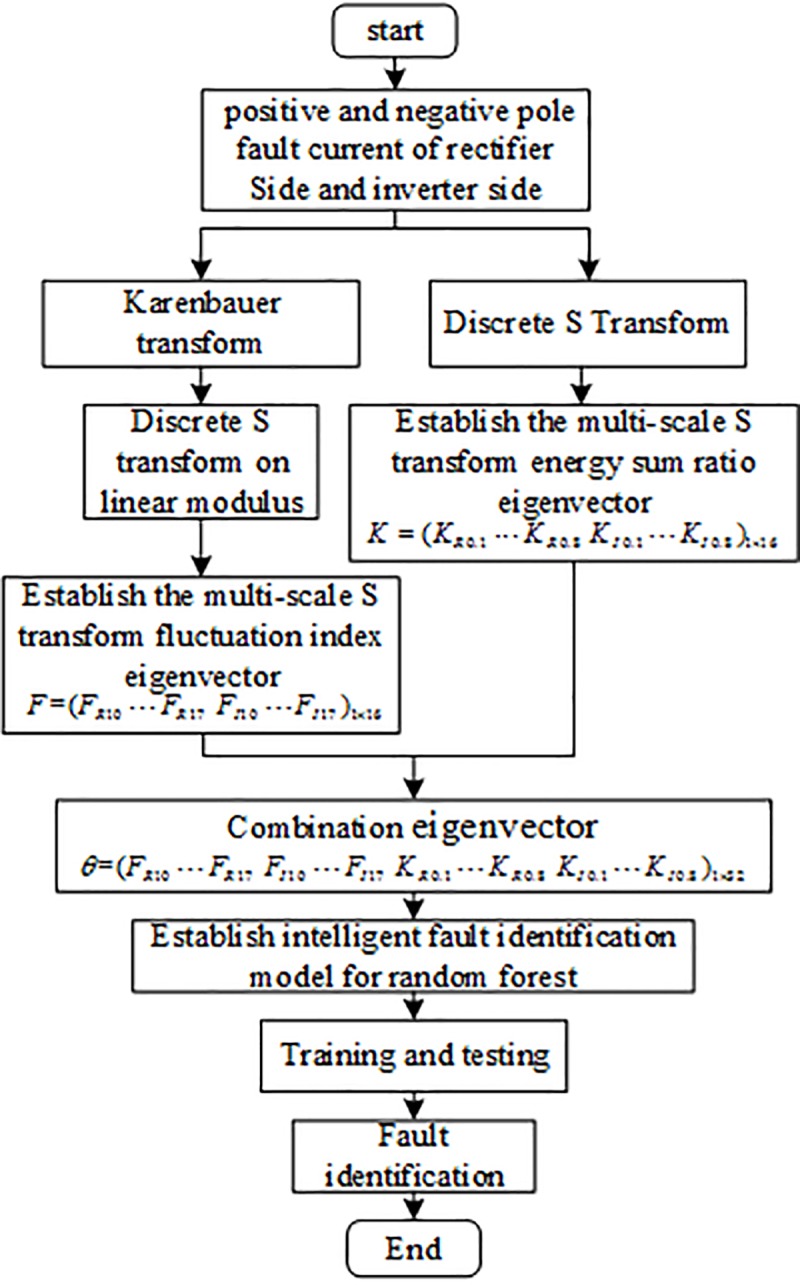
Intelligent fault identification algorithm flow.

The implementation steps of the intelligent fault identification algorithm are as follows:

1. Extract fluctuation index features
  1. Perform Karenbauer transform on current fault traveling wave;
  2. Perform discrete S transform on linear modulus;
  3. Select the fault traveling wave signals of 8 characteristic frequencies *f*_*l*_(*l* = 10,11,12,13,14,15,16,17)kHz after S transform, and calculate the fluctuation index of 80 sampling data in the 2ms time window of the fault traveling wave at each frequency, and then the internal and external fault identification eigenvector *K* = (*K*_*R*0.1_⋯*K*_*R*0.8_ *K*_*I*0.1_⋯*K*_*I*0.8_)_1×16_ based on the multi-scale S transform fluctuation index is obtained.
2. Extract energy sum ratio features
  1. Perform discrete S transform on the positive and negative current signals;
  2. Select the fault traveling wave signals of 8 characteristic frequencies *f*_*j*_(*j* = 0.1,0.2,0.3,0.4,0.5,0.6,0.7,0.8)kHz after S transform, calculate the energy and ratio of 80 samples of the positive and negative fault current traveling wave within 2ms at each frequency, and then obtain the fault pole identification characteristic vector of multi-scale S transform energy and ratio *K* = (*K*_*R*0.1_⋯*K*_*R*0.8_ *K*_*I*0.1_⋯*K*_*I*0.8_)_1×16_.
3. Combine the internal and external fault identification eigenvector *F* = (*F*_*R*11_⋯*F*_*R*17_ *F*_*I*11_⋯*F*_*I*17_)_1×16_ based on S-transform multi-scale fluctuation index with the fault pole identification eigenvector *K* = (*K*_*R*0.1_⋯*K*_*R*0.8_ *K*_*I*0.1_⋯*K*_*I*0.8_)_1×16_ based on S-transformed multi-scale energy sum ratio to form a combined eigenvector *θ* = (*F*,*K*) = (*F*_*R*10_⋯*F*_*R*17_ *F*_*I*10_⋯*F*_*I*17_ *K*_*R*0.1_⋯*K*_*R*0.8_ *K*_*I*0.1_⋯*K*_*I*0.8_)_1×32_ that can reflect both internal and external faults characteristics and the fault pole characteristics, and thus to characterize the fault characteristics of the HVDC transmission line.
4. Label each sample eigenvector as the sample data of the random forest, and then put the training samples into the random forest for training, and finally a random forest intelligent fault identification model is obtained. The test sample is input into the trained random forest intelligent fault identification model to obtain the identification result. The fault identification algorithm flow is shown in Fig 9.

## 6 Simulation

**Fig 10:**
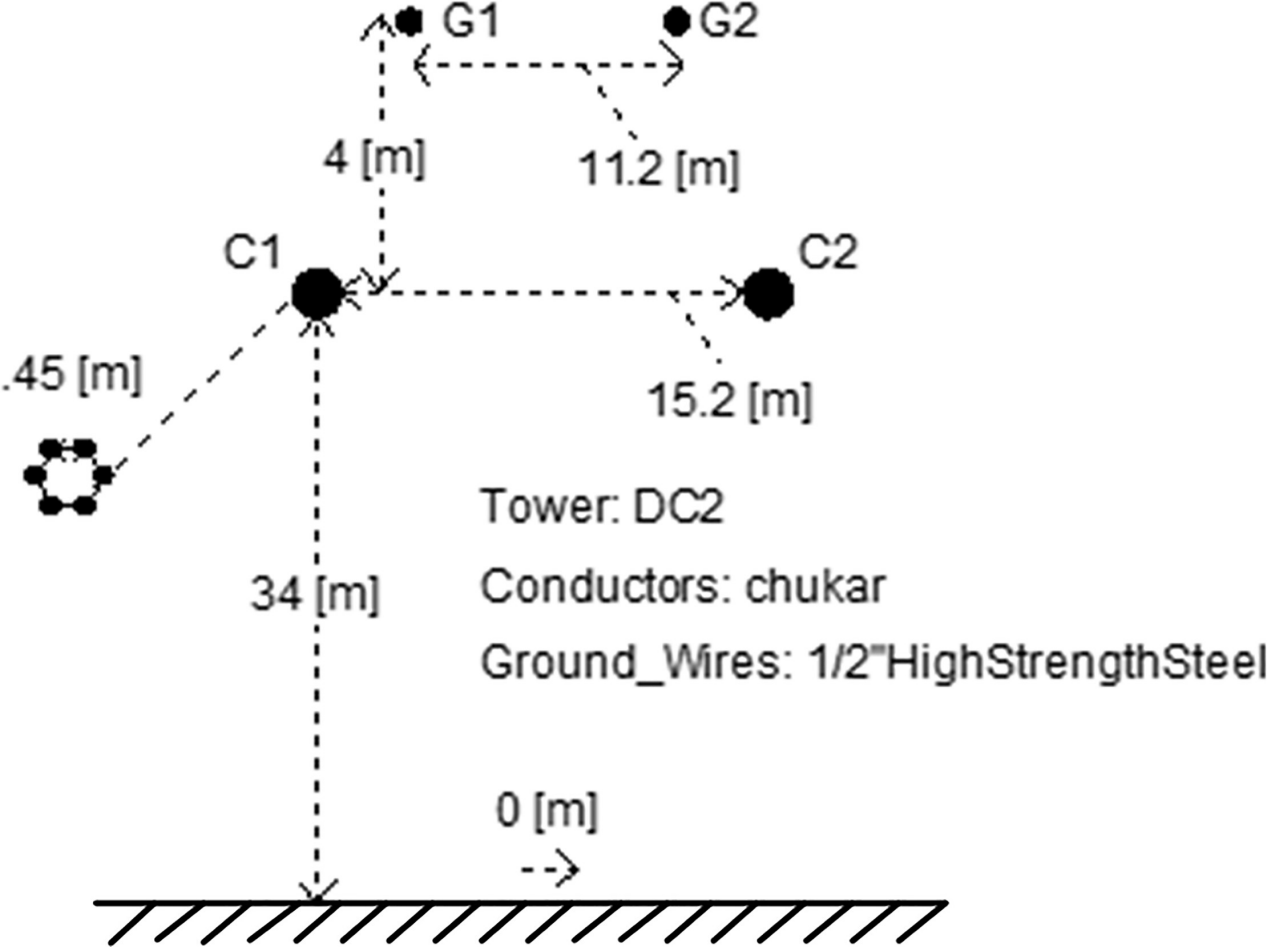
DC line DC2 tower.

The test platform parameters of the experiments in this paper are shown in Table 2. The simulation model of ±500kV HVDC transmission system shown in Fig 1 is established in PSCAD/EMTDC. The parameters of the model refer to the Three Gorges-Changzhou HVDC transmission project. The transmission power is 3000MW, the rated voltage is 500kV, and the rated current is 3kA. The transmission line model uses a frequency-dependent model. The line structure uses a DC2 tower. The tower parameters refer to the G1 tower type used in the project [8]. The DC2 tower of the DC line is shown in Fig 10. The length of the transmission line is set to 1000km, and the parameters of the DC transmission line are shown in Table 3.

**Table 2:** Test platform parameters.

Term	Parameters
system version	Windows 10 Professional 64-bit
Processor model (CPU)	Pentium(R) Dual-Core E6700 @
Processing speed	3.2GHz
Memory (RAM)	2GB

**Table 3:** DC transmission line parameters.

Line type	parameters	Value and unit
wire	Wire radius	0.0203454 m
	DC Resistance	0.02275Ω/km
Ground wire	Ground wire radius	0.0055245m
	DC Resistance	2.8645Ω/km
	resistivity	100Ω·m

The boundary of the DC transmission line has an obvious attenuation effect on the high-frequency components (10 kHz and above) in the fault transient signal [6,7,22]. In order to improve the performance of the protection algorithm, this paper uses the multi-scale S-transform fluctuation index to realize the internal and external fault identification, and the frequency range of the signal after S transform is 10 kHz to 17 kHz. According to the Nyquist sampling theorem, in order to recover the signal without distortion, the sampling frequency should not be less than twice the highest frequency in the signal spectrum, so the sampling frequency of the proposed protection algorithm should not be less than 34 kHz.

**Fig 11:**
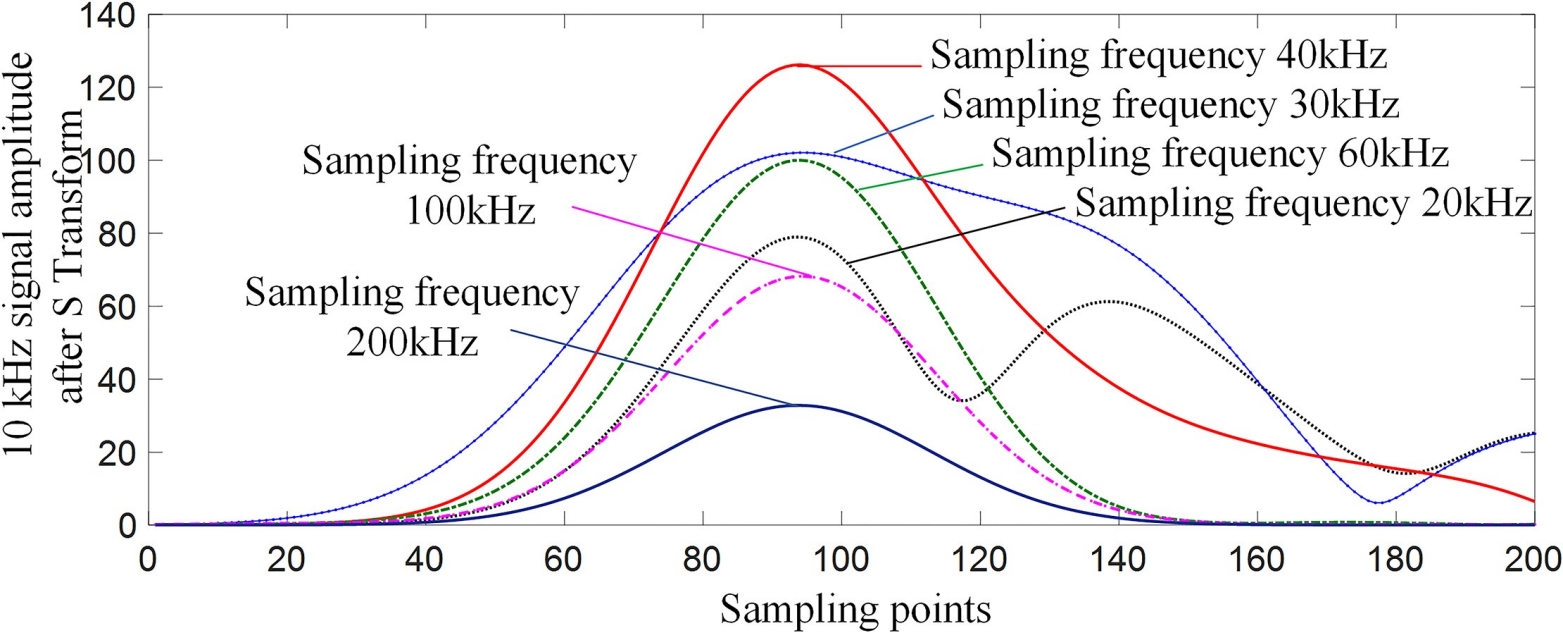
Amplitude comparison of 10 kHz frequency component signals after S-transform at different sampling frequencies.

In the HVDC transmission system shown in Fig 1, when a ground fault occurs at a point of *F*_3_ (fault distance 500 km, transitional resistance 10 Ω), the same frequency components (10 kHz) after S-transform at different sampling frequencies (20 kHz, 30 kHz, 40 kHz, 60 kHz, 100 kHz, 200 kHz) are compared, and the signal conversion results are shown in Fig 11. The analysis of Fig 11 shows that in the sampling frequency range from 40 to 200 kHz, the lower the sampling frequency is, the larger the amplitude of the 10 kHz frequency component signal after S-transform is, and the more obvious the characteristic difference of the fault signal is. When the sampling frequency is less than 40 kHz, the waveform change of the S-transformed waveform is not obvious, and the corresponding signal amplitude at the 10 kHz frequency is less than the signal amplitude at the sampling frequency of 40 kHz. Therefore, in order to better reflect the change characteristics of the fault, the sampling frequency is chosen to be 40 kHz in this paper.

The sampling data in the 2ms time window after the fault are selected to calculate the fluctuation index and the energy sum ratio, and the fluctuation index eigenvector *F* = (*F*_*R*10_⋯*F*_*R*17_ *F*_*R*10_⋯*F*_*R*17_)_1×16_ and the energy sum ratio eigenvector *K* = (*k*_*R*0.1_⋯*k*_*R*0.8_ *K*_*I*0.1_⋯*K*_*I*0.8_)_1×16_ are obtained. And the combined eigenvector of each sample is *θ* = (*F*,*K*) = (*F*_*R*10_⋯*F*_*R*17_ *F*_*I*10_⋯*F*_*I*17_ *K*_*R*0.1_⋯*K*_*R*0.8_ *K*_*I*0.1_⋯*K*_*I*0.8_)_1×32_. Therefore, the input dimension of each sample is 1×32, the dimension of the sample input set is 1×32×*N*, and *N* is the total number of samples in the sample set.

In order to verify the performance of the algorithm, this paper selects the simulation experiment of the HVDC transmission system under different fault types, different transitional resistances and different fault distances.

### 6.1 Establishment of intelligent fault identification model for random forests

The training samples of random forests are composed of two parts: the sampling data free from noise interference and the sampling data affected by noise when different faults occur in the HVDC transmission system. In this paper, the internal faults include 3 cases including positive pole fault (LPG), negative pole fault (LNG) and short-circuit fault between positive and negative poles(LPN), considering a total of 11 cases of the fault distances (from the rectifier side protection installation) 1km, 100km, 200km, 300km, 400km, 500km, 600km, 700km, 800km, 900km, 999km, and considering a total of 8 cases of the transitional resistances 1Ω, 10Ω, 100Ω, 200Ω, 300Ω, 400Ω, 500Ω, 600Ω. Thus, there are a total of 3×11×8 = 264 internal fault data samples that are insusceptible to noise. Considering the external faults, including positive and negative pole faults (RPG, RNG) on the rectifier side and positive and negative pole faults (IPG, ING) on the inverter side, the transitional resistance is set in the same way as it is in the zone, so there are a total of 4×8 = *32* external data samples that are insusceptible to noise interference. The sampling of external noise interference data samples are consistent with that of external non-noise interference data samples, which are 4×8 = *32* as well. Therefore, the total number of training samples in this paper is 3×11×8+4×32+4×32 = 328.

**Fig 12:**
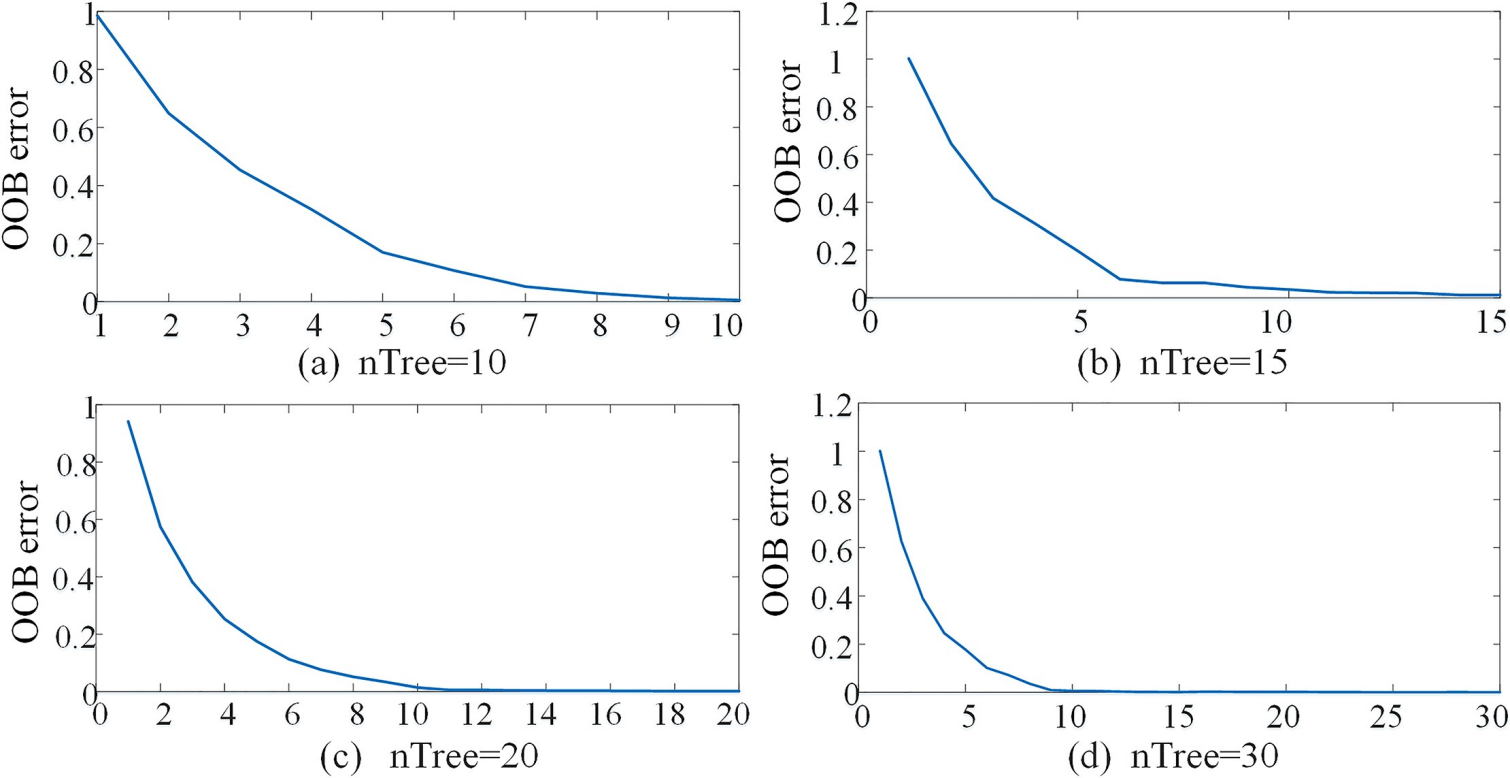
OOB error curve.

In order to verify the accuracy and reliability of the random forest model, Leo Breiman experimentally proved that the OOB estimation is an unbiased estimation of the generalization error of RF [24]. The smaller the OOB error value, the better the generalization performance of the RF algorithm. Therefore, in this paper, the out-of-bag data error rate (OOB error) is used as a performance index to evaluate the generalization error of the RF algorithm. The training sample of fault features is used as a test sample to be input into the trained random forest intelligent fault identification model for testing. The OOB error curve of the training sample is shown in Fig 12. It can be known from Fig 12 that when nTree = 10 or nTree = 15, as the number of trees in the random forest increases, the OOB error curve gradually decreases; when nTree = 20, the OOB error has stabilized. Because when the model already has excellent generalization performance, random forests will not produce overfitting as more trees are added, but will generate limit values for generalization errors [24]. Therefore, as shown in Fig 12, When nTree> 20, OOB error has not decreased with the increase of random forest trees. Therefore, the number of optimal decision trees is 20 in this paper. At this time, the intelligent fault identification model based on random forest can identify HVDC transmission line faults accurately.

### 6.2 Analysis of training sample identification results

**Fig 13:**
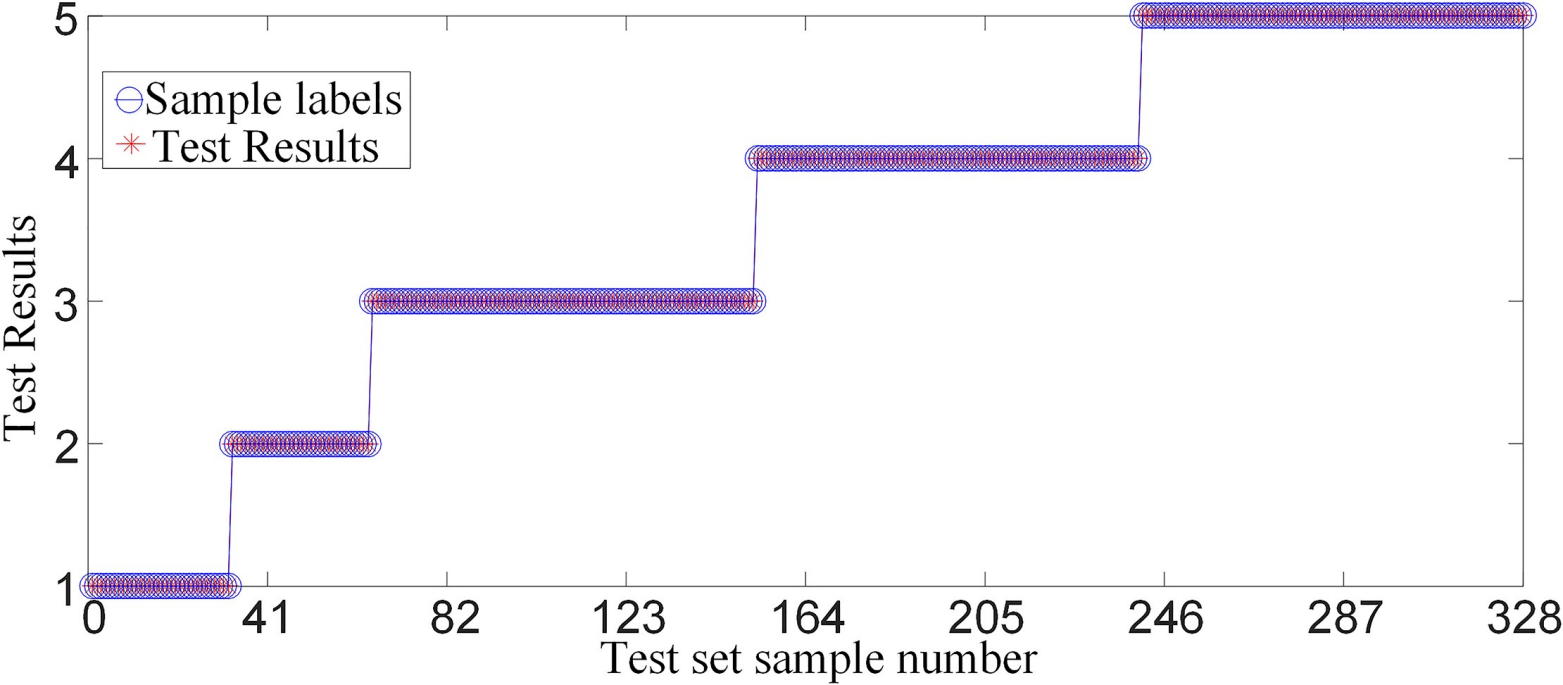
Comparison of test results when the training set is used as a test set.

Fig 13 shows a comparison of the test results when the training sample is used as a test sample. It can be seen from Fig 13 that the training samples selected in this paper can all be correctly identified in the random forest intelligent fault identification model.

### 6.3 Analysis of test sample identification results

The fault characteristic test samples of different fault types, different transitional resistances and different fault distances are fed into the intelligent fault identification model of HVDC transmission line for fault identification, and the test results are analyzed.

#### 6.3.1 Analysis of test results when different types of faults occur

**Fig 14:**
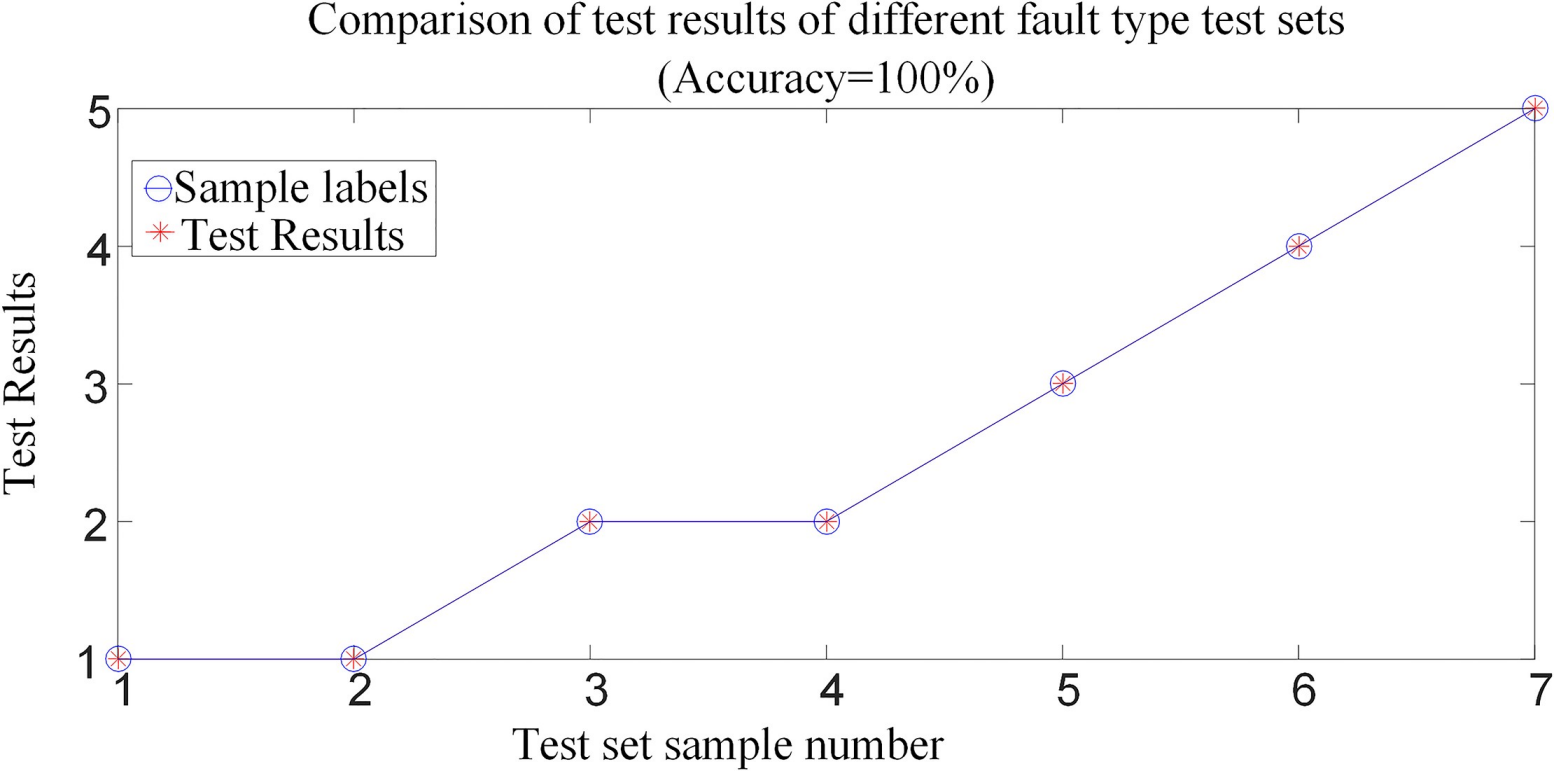
Comparison of test results when different types of faults occur.

In order to verify the adaptability of the protection algorithm for different fault types, a total of 7 samples were tested under different fault types in the system shown in Fig 1, in which the positive and negative poles are fault at *F*_1_ and *F*_2_ outside the rectification side area, the transmission line are fault at *F*_3_, *F*_4_ and *F*_5_, and the positive and negative poles are fault at *F*_6_ and *F*_7_ outside the inverter side, respectively. In the case of the same fault distance and transitional resistance, the test sample sets constructed with different fault type samples are input into the trained random forest model for testing. Comparison of the test results when different types of faults occur is shown in Fig 14, and the corresponding simulation verification results of corresponding fault conditions are shown in Table 4.

**Table 4:** Simulation verification results when different types of faults occur.

Fault types	Fault distance (from rectifier lateral protection installation km)	Transitional Resistors (Ω)	Category Label	Identify results	
				Output label	Fault types
RPG(*F*_1_)	-	350	1	1	External positive-pole
IPG(*F*_6_)	-	350	1	1	
RNG(*F*_2_)	-	350	2	2	External negative-pole
ING(*F*_7_)	-	350	2	2	
LPG(*F*_3_)	150	350	3	3	Internal positive-pole
LNG(*F*_4_)	150	350	4	4	Internal negative-pole
LPN(*F*_5_)	150	350	5	5	Two-poles short circuit

Table 4 and Fig 14 show that the fault identification model is not affected by the fault type of the HVDC transmission line, and can achieve accurate internal and external fault identification and fault poles selection.

#### 6.3.2 Analysis of test results when different transitional resistance faults occur

**Fig 15:**
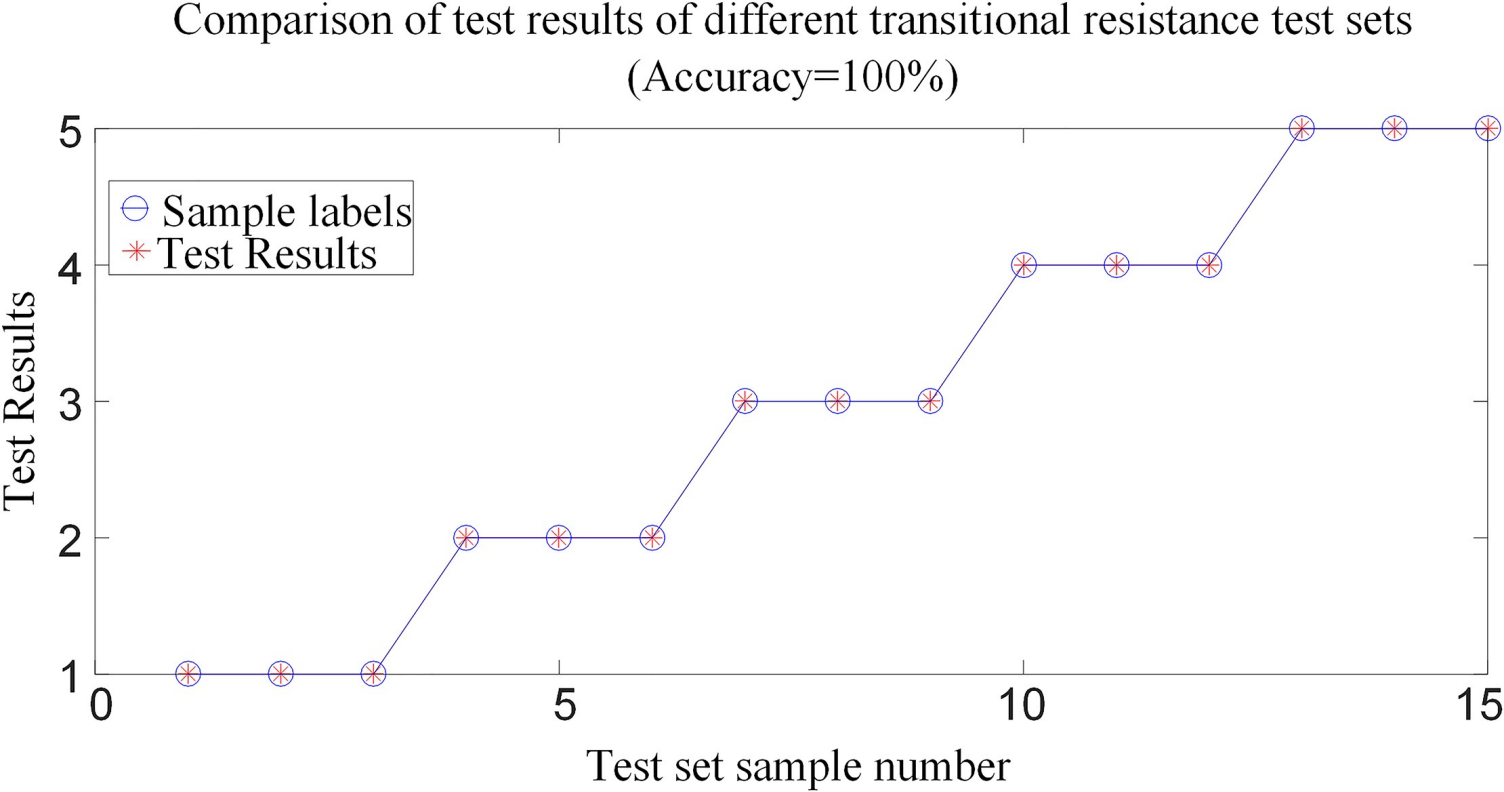
Comparison of test results when faults with different transitional resistances occur.

To verify the performance of the protection algorithm for different transitional resistances, especially for remote high-impedance faults on the line. It is supposed that the positive and negative poles are fault at *F*_1_ outside the rectification side area, the transmission line are fault at *F*_3_, *F*_4_ and *F*_5_, and the positive and negative poles are fault at *F*_7_ outside the inverter side of the system shown in Fig 1. Select 15 test samples to build a test sample set and input the trained random forest model for testing. Comparison of test results when different transitional resistance of faults occur is shown in Fig 15, and Table 5 shows the simulation verification results for the corresponding fault conditions.

**Table 5:** Simulation verification results when faults with different transitional resistances occur.

Fault types	Fault distance (from rectifier lateral protection installation km)	Tran-sitional Resistors (Ω)	Cate-gory Label	Identify results	
				Output label	Fault types
RPG(*F*_1_)	-	150	1	1	External positive-pole
		450	1	1	
		600	1	1	
ING(*F*_7_)	-	150	2	2	External negative-pole
		450	2	2	
		600	2	2	
LPG(*F*_3_)	10	150	3	3	Internal positive-pole
		450	3	3	
		600	3	3	
LNG(*F*_4_)	999	150	4	4	Internal negative-pole
		450	4	4	
		600	4	4	
LPN(*F*_5_)	450	150	5	5	Two-poles short circuit
		450	5	5	
		600	5	5	

Table 5 and Fig 15 show that in the case of different transitional resistances, the model can perform accurate fault identification and fault selection in and outside the area, and has a strong ability to withstand transitional resistance. Especially in the case of high-impedance faults at the far end of the transmission line, the model can also perform accurate fault identification and fault pole selection.

#### 6.3.3 Comparative analysis of test results when failure occurs at different distances

**Fig 16:**
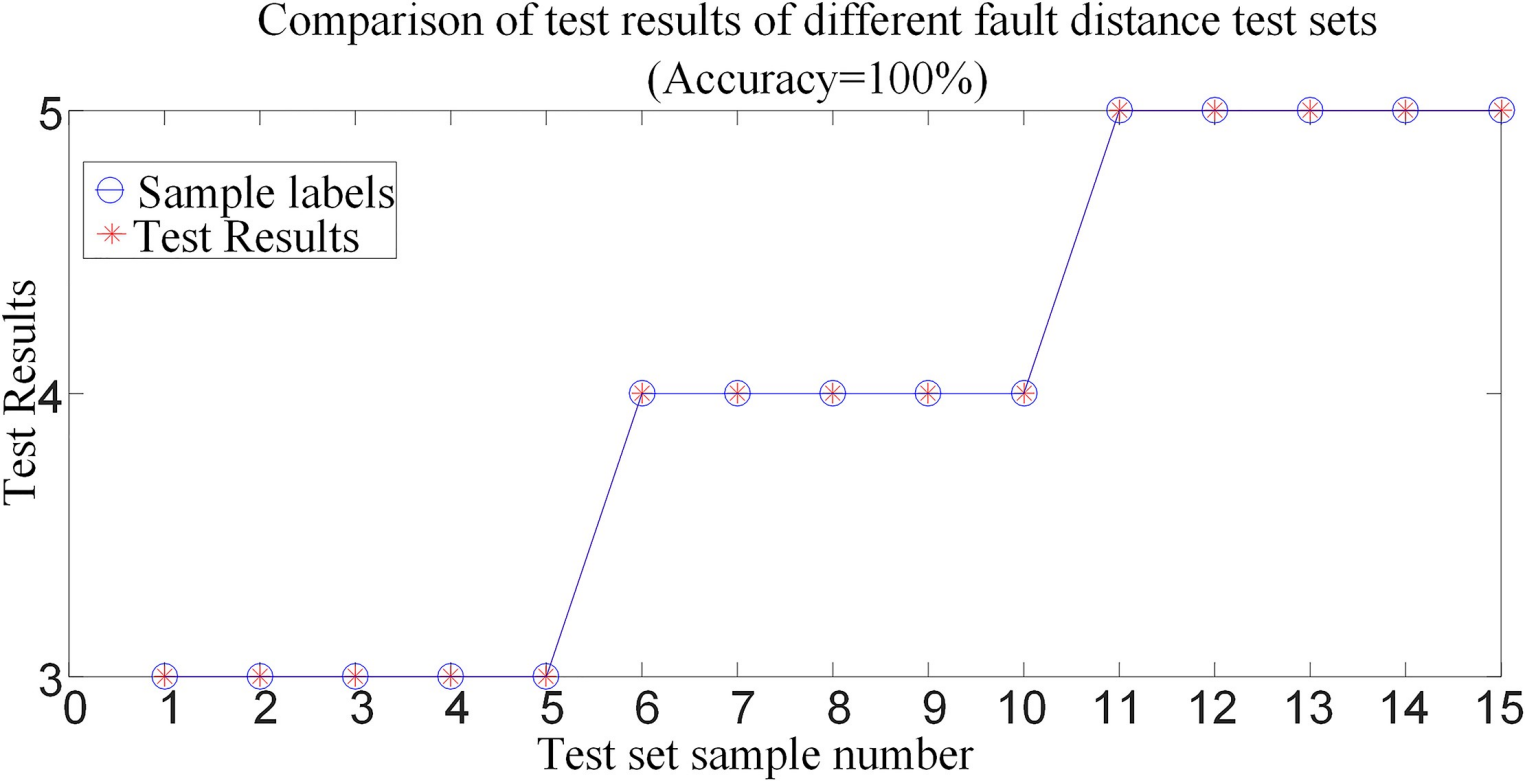
Comparison of test results when faults occur at different distances.

To verify the performance of the protection algorithm for different fault distances, it is supposed that the transmission line are fault at *F*_3_, *F*_4_ and *F*_5_ of the system shown in Fig 1. Select 15 test samples to build a test sample set and input the trained random forest model for testing. The test results are compared as shown in Fig 16. Table 6 shows the simulation verification results for the corresponding fault conditions.

**Table 6:** Simulation verification results when faults occur at different distances.

Fault types	Tran-sitional Resistors (Ω)	Fault distance (from rectifier lateral protection installation km)	Cate-gory Label	Identify results	
				Output label	Fault types
LPG(*F*_3_)	600	10	3	3	Internal positive-pole
		150	3	3	
		250	3	3	
		350	3	3	
		450	3	3	
LNG(*F*_4_)	600	650	4	4	Internal negative-pole
		750	4	4	
		850	4	4	
		950	4	4	
		990	4	4	
LPN(*F*_5_)	600	50	5	5	Two-poles short circuit
		250	5	5	
		450	5	5	
		650	5	5	
		850	5	5	

Table 6 and Fig 16 show that the fault identification model is not affected by the fault distance, and accurate fault identification and fault pole selection can be achieved under different fault distances.

As can be seen from Table 4 to Table 6 and Fig 14 to Fig 16, within 2ms after the fault occurs, the intelligent fault identification model based on random forests for HVDC transmission line is insusceptible to fault types and fault distances, and the algorithm uses the same network to realize both internal and external fault identification and fault pole selection, with strong resistance to transitional resistances.

### 6.4 Performance analysis of protection algorithms

As is known, in the existing HVDC transmission line protection, although the traveling wave protection speed is fast, there is always a problem of low reliability. The main reason is that under complicated operating conditions, the noise interference and high resistance fault make the transient traveling wave signal obtained by the protection unit weak, which makes it difficult to extract wavefront information. So using only the wavefront information of the fault traveling wave will lead to the decrease of protection reliability. At the same time, for HVDC transmission line protection using only travelling wave peak information, the protection will fail when data distortion or peak information is lost. In order to overcome the shortcomings of the above traditional protection, this paper constructs the intelligent fault identification model based on random forests for HVDC transmission lines. The following is an analysis of the performance of the protection algorithm considering data loss and noise interference.

#### 6.4.1 Performance analysis of protection algorithm in case of data loss

**1**. **Performance test of protection algorithm when data is lost near the fault wave head**

**Fig 17:**
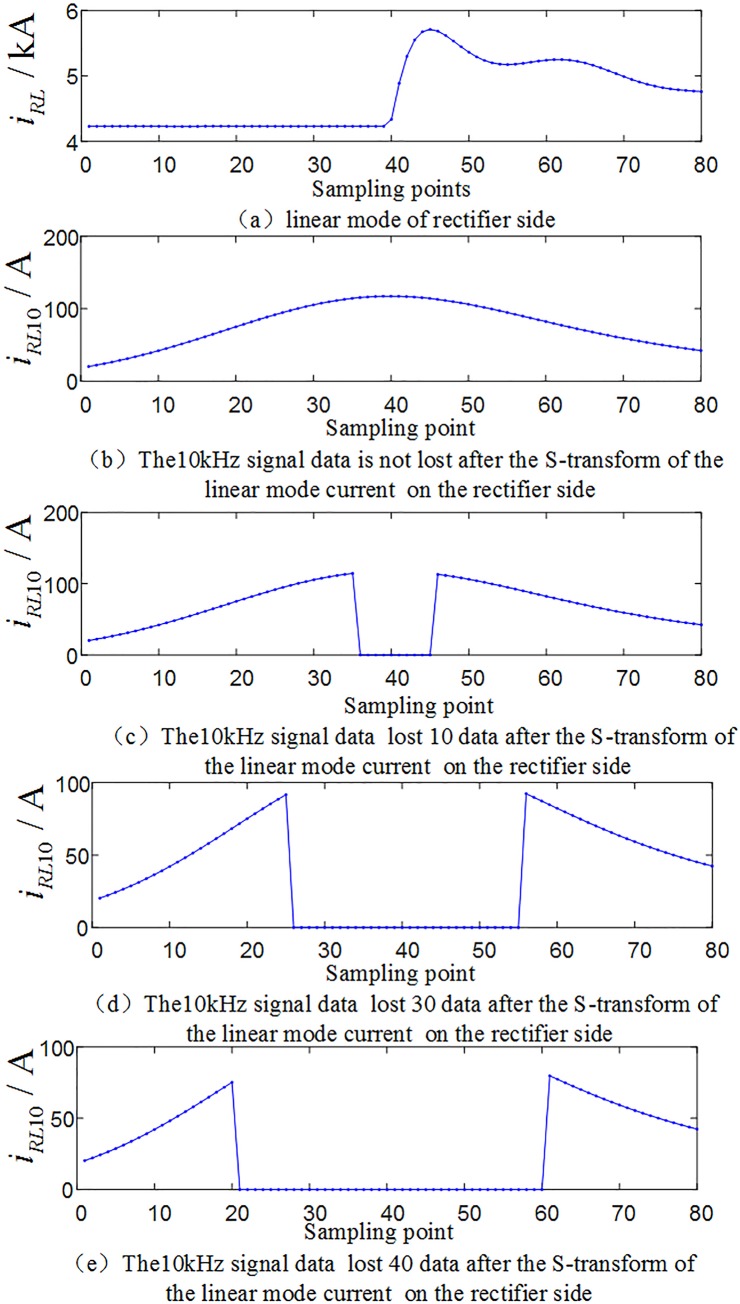
Correlation waveforms of data loss (10~40 data) near the traveling wave head.

In order to verify the performance of the protection algorithm in the case of data loss near the fault wave head, it is supposed that the positive and negative poles are fault at *F*_1_ outside the rectification side area, the transmission line is fault at *F*_3_, *F*_4_ and *F*_5_, and the positive and negative poles are fault at *F*_7_ outside the inverter side, and test the faults in the system shown in Fig 1, respectively. At the characteristic frequency of 10 kHz, considering 4 cases of information loss of 10, 20, 30, 40 sampling data near the wavefront of the current traveling wave, and 5×4 = 20 sets of test sample of data loss near the wavefront were obtained. Fig 17 shows the waveform of the loss of data near the wavefront of the traveling wave when the transmission line is fault at *F*_3_ (transitional resistance is 10Ω and *F*_3_ is at a distance of 500km from the rectifier side protection installation).

**Fig 18:**
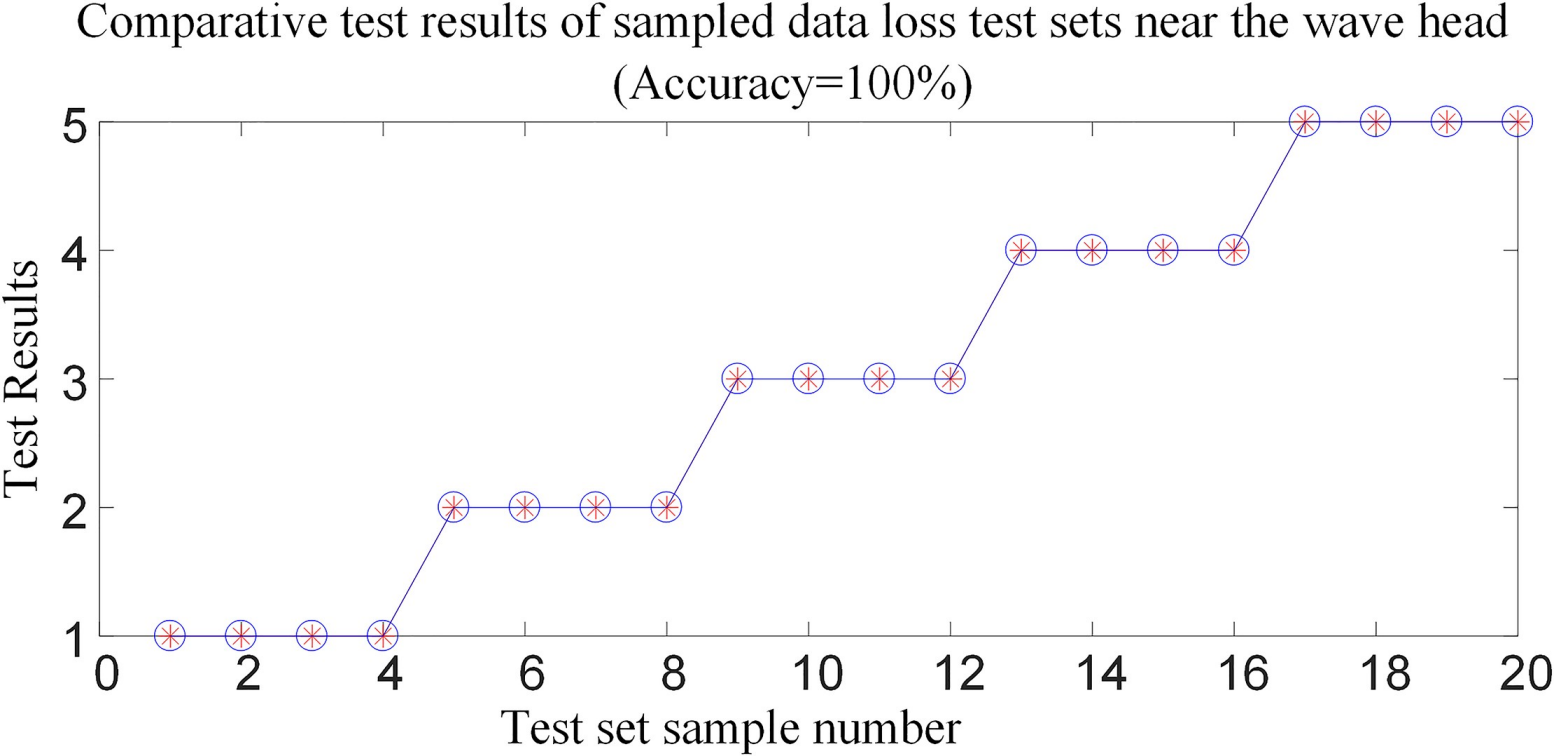
Sample data loss near the traveling wave head (10~40 data) recognition result.

The above 20 sets of test samples with data loss are input into the random forests intelligent fault identification model for testing. The test results are compared as shown in Fig 18, and Table 7 shows the simulation verification results for the corresponding fault conditions.

**Table 7:** Performance test of protection algorithm when data is lost near the fault wave head.

Fault types	Fault distance (km)	Transitional Resistors(Ω)	Number of data loss of peak value	Category Label	Identify results	
					Output label	Fault types
RPG(*F*_1_)	-	100	10	1	1	External positive-pole
			20	1	1	
			30	1	1	
			40	1	1	
ING(*F*_7_)	-	200	10	2	2	External negative-pole
			20	2	2	
			30	2	2	
			40	2	2	
LPG(*F*_3_)	1	400	10	3	3	Internal positive-pole
			20	3	3	
			30	3	3	
			40	3	3	
LNG(*F*_4_)	500	500	10	4	4	Internal negative-pole
			20	4	4	
			30	4	4	
			40	4	4	
LPN(*F*_5_)	999	600	10	5	5	Two-poles short circuit
			20	5	5	
			30	5	5	
			40	5	5	

**2**. **Protection algorithm performance test when sampling point data is randomly lost**

**Fig 19:**
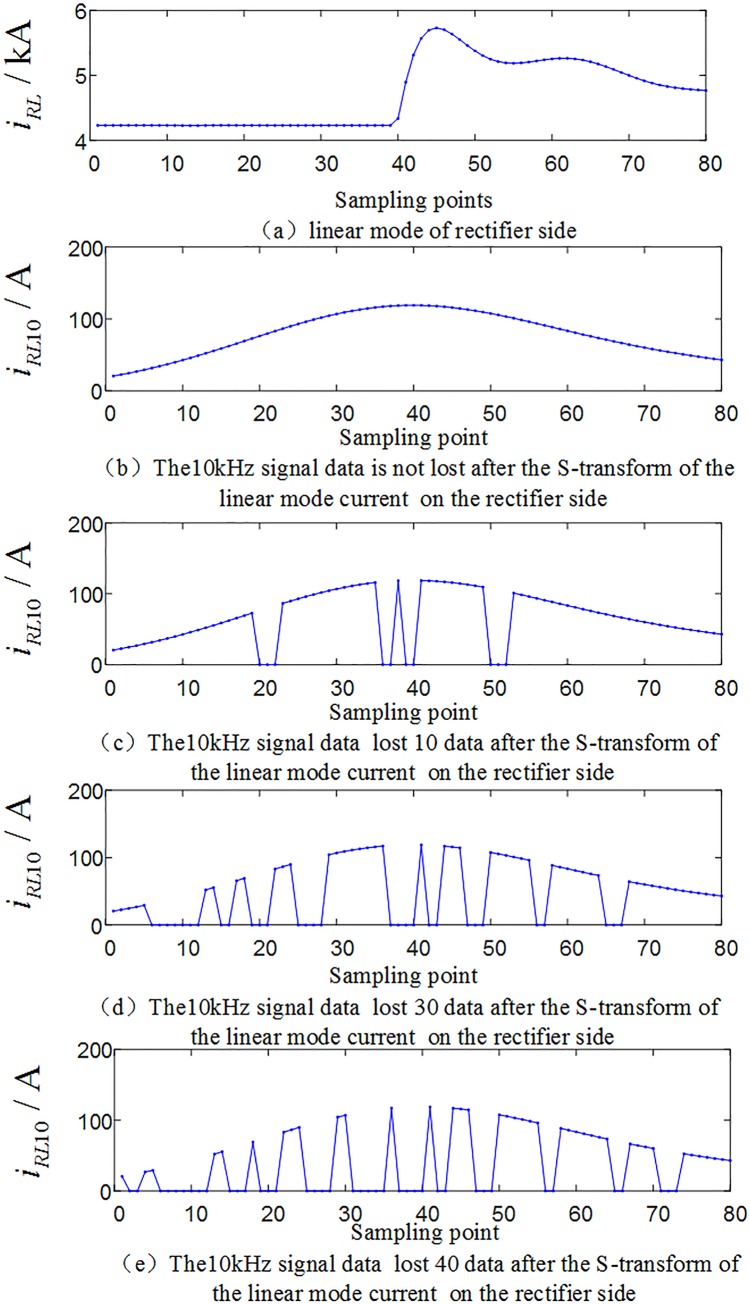
Correlation waveforms of data loss (10~40 data) randomly.

In order to verify the performance of the protection algorithm in the case of random loss of traveling wave data, set the positive and negative poles are fault at *F*_1_ outside the rectification side area, the transmission line are fault at *F*_3_, *F*_4_ and *F*_5_, and the positive and negative poles are fault at *F*_7_ outside the inverter side, and test the faults in the system shown in Fig 1, respectively. At the characteristic frequency of 10 kHz, considering 4 cases of information randomly loss of 10, 20, 30, 40 sampling data within 2ms, and a total of 5×4 = 20 sets of test samples with random loss of traveling wave data are obtained. Fig 19 shows the corresponding waveform of the random loss of the fault traveling wave data when the transmission line is fault at *F*_4_ (transitional resistance is10Ω, and *F*_4_ is 500km away from the rectifier side protection installation).

**Fig 20:**
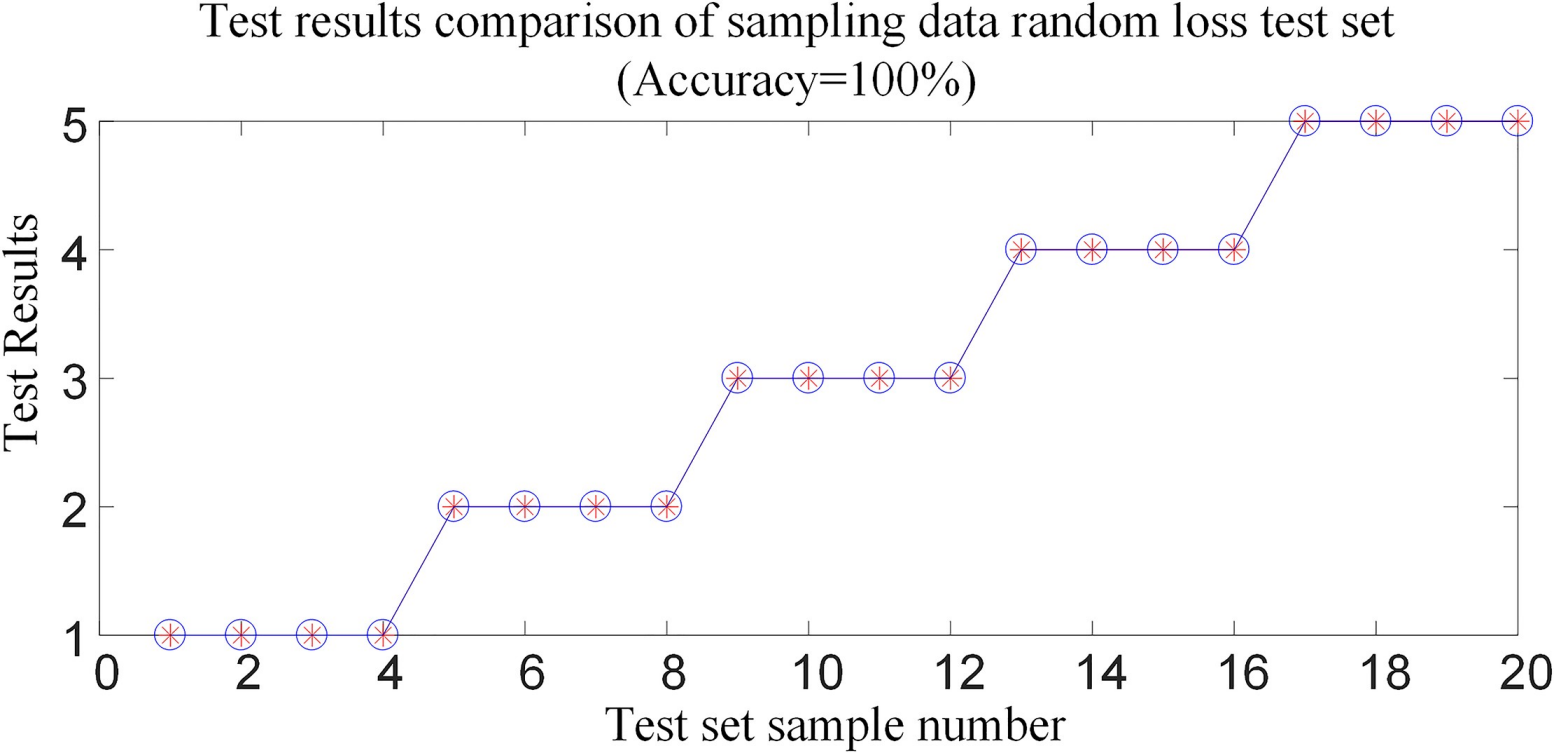
Sample data loss randomly (10~40 data) recognition result.

The above 20 sets of test samples with data loss randomly are input into the random forests intelligent fault identification model for testing. The test results are compared as shown in Fig 20, and Table 8 shows the simulation verification results for the corresponding fault conditions.

**Table 8:** Protection algorithm performance test when sampling point data is randomly lost.

Fault types	Fault distance (km)	Transi-tional Resistors(Ω)	Number of data loss randomly	Category Label	Identify results	
					Output label	Fault types
RPG(*F*_1_)	-	100	10	1	1	External positive-pole
			20	1	1	
			30	1	1	
			40	1	1	
ING(*F*_7_)	-	200	10	2	2	External negative-pole
			20	2	2	
			30	2	2	
			40	2	2	
LPG(*F*_3_)	1	400	10	3	3	Internal positive-pole
			20	3	3	
			30	3	3	
			40	3	3	
LNG(*F*_4_)	500	500	10	4	4	Internal negative-pole
			20	4	4	
			30	4	4	
			40	4	4	
LPN(*F*_5_)	999	600	10	5	5	Two-poles short circuit
			20	5	5	
			30	5	5	
			40	5	5	

The analysis of Table 7~Table 8 and Fig 17~Fig 20 show that in the case of partial wave head data loss or partial data random loss (10~40 data), the proposed algorithm can still realize fault identification and fault poles selection.

The algorithm calculates the fluctuation index of the sampling data within 2ms time after the fault occurs, establishes the characteristic sample set by using the fluctuation index of the multi-scale signal, and identifies the fault through random forest intelligent fault identification model. Even if a certain scale signal is lost, the characteristics of other scale signals can still play the role of fault identification, so the impacts of sample value data loss and weak traveling wave signal can be reduced to some extent. According to theoretical analysis and simulation results, the proposed algorithm is insusceptible to data loss of sampling points.

#### 6.4.2 Protection algorithm performance test considering noise interference

**Fig 21:**
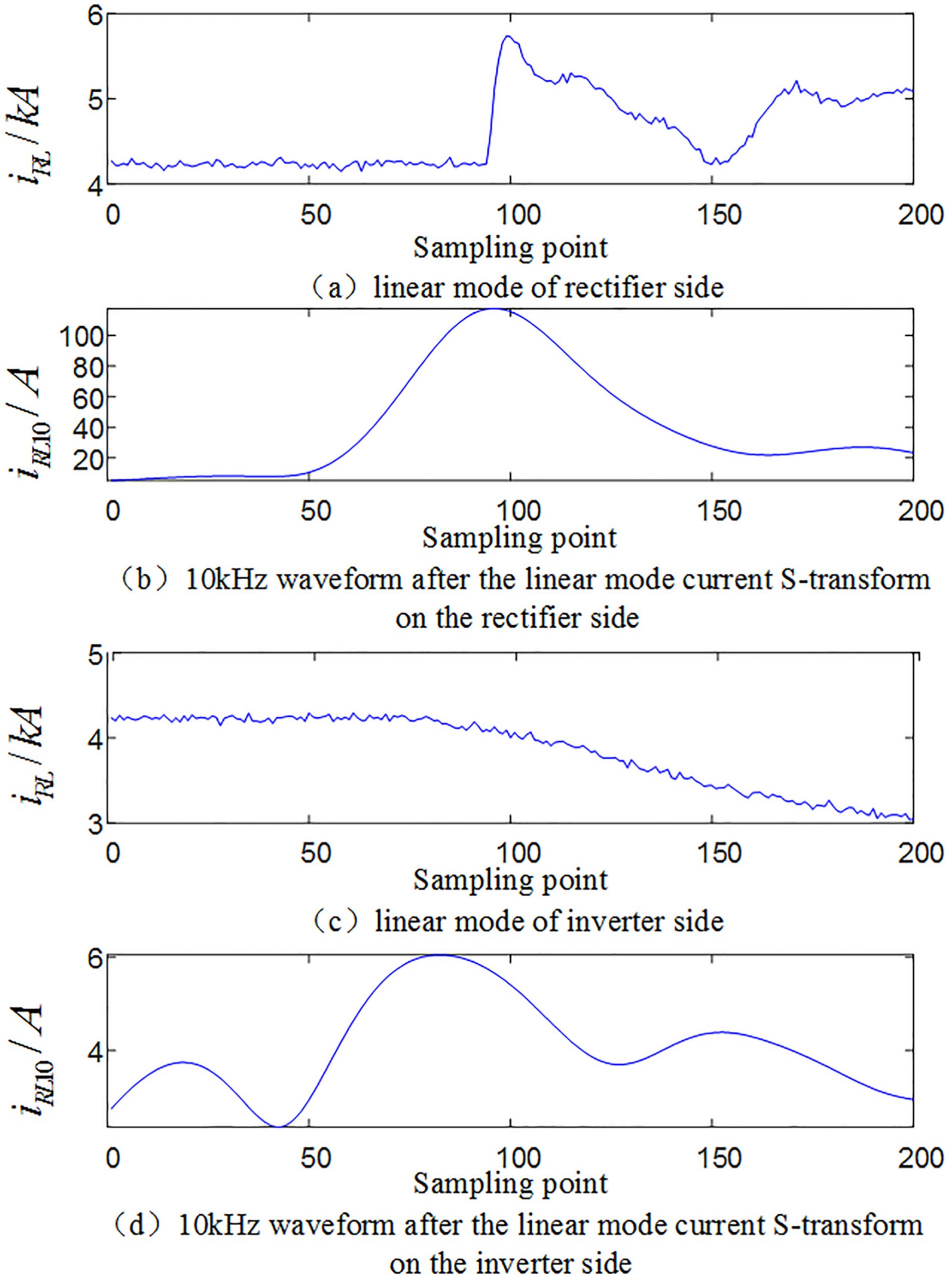
Correlation waveforms in the case of SNRs = 30dB.

In order to verify the performance of the protection algorithm under the impact of noise, set the positive pole is fault at *F*_6_ outside the inverter side, the transmission lines are fault at *F*_3_, *F*_4_ and *F*_5_, and the negative pole is fault at *F*_2_ outside the rectification side area, were tested under different fault types in the system shown in Fig 1, respectively. Considering three cases of noise interference, SNRs = 30, 40, 50db, respectively, and obtains 5×3 = 15 groups of noise interference test samples. Fig 21 shows the corresponding waveform in the case of SNRs = 30dB when the transmission line is fault at *F*_3_ (transitional resistance is10Ω, and *F*_3_ is 500km away from the rectifier side protection installation)and the positive and pole is fault at *F*_1_ (transitional resistance is10Ω) outside the rectification side.

**Fig 22:**
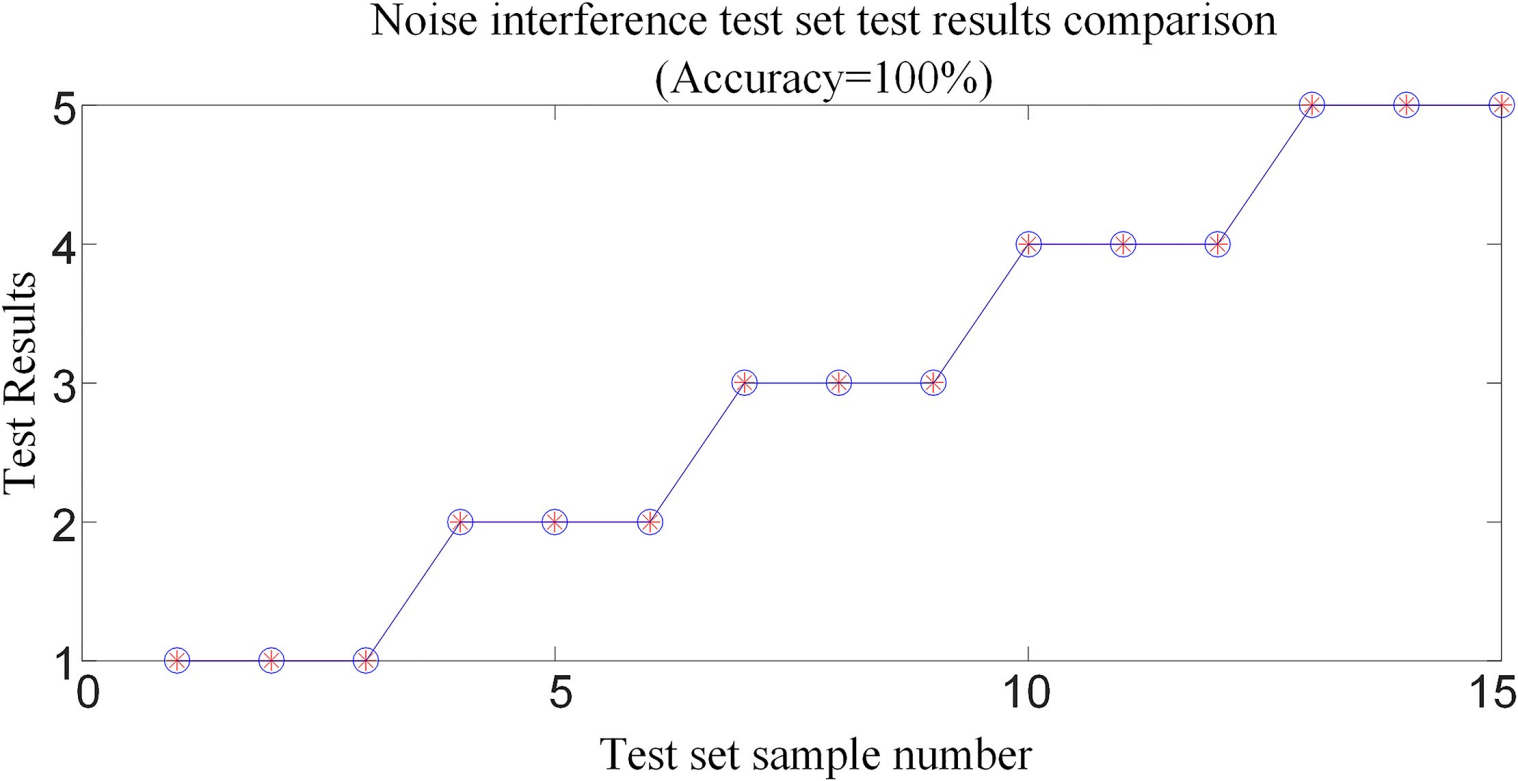
30~50dB noise interference test results.

The above 15 sets of test samples with noise interference are input into the random forests intelligent fault identification model for testing. The test results are compared as shown in Fig 22, and Table 9 shows the simulation verification results for the corresponding fault conditions.

**Table 9:** Protection algorithm performance test considering noise interference.

Fault types	Fault distance (km)	Transitional Resistors(Ω)	SNRs(dB)	Category Label	Identify results	
					Output label	Fault types
IPG (*F*_6_)	-	100	30	1	1	External positive-pole
			40	1	1	
			50	1	1	
RNG (*F*_2_)	-	200	30	2	2	External negative-pole
			40	2	2	
			50	2	2	
LPG (*F*_3_)	999	400	30	3	3	Internal positive-pole
			40	3	3	
			50	3	3	
LNG (*F*_4_)	500	500	30	4	4	Internal negative-pole
			40	4	4	
			50	4	4	
LPN (*F*_5_)	1	600	30	5	5	Two-poles short circuit
			40	5	5	
			50	5	5	

It can be seen from Table 9 and Fig 22 that the intelligent fault identification model can still realize fault identification when the remote high-resistance fault is affected by noise, and can reliably identify the internal and external faults and make fault pole selection even when the signal-to-noise ratio is 30dB. Therefore, the protection algorithm in this paper is insusceptible to noise interference and has a strong anti-noise ability.

#### 6.4.3 Quick-action discussion

**Fig 23:**
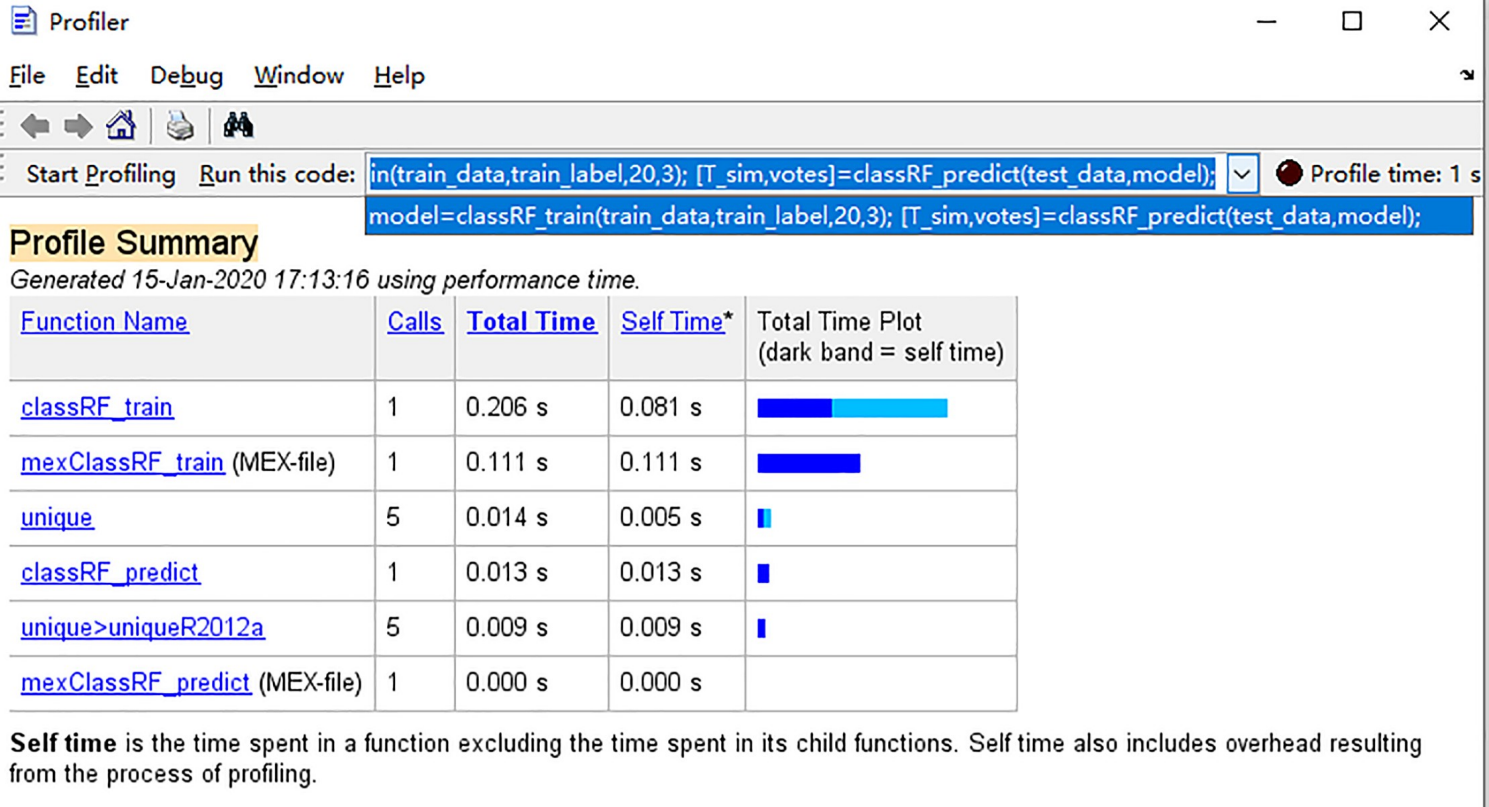
Sample test time.

Limited by laboratory conditions, this paper uses the test platform shown in Table 2 to study the rapidity. The protection action time mainly includes algorithm time and channel transmission time. In terms of algorithm time, the intelligent algorithm uses a data window length of 2ms, and only requires simple multiplication and accumulation operations, with a small amount of calculation. With the computing power of the existing DSP, the computing time does not exceed 0.5ms[26]. After the intelligent model is trained, it does not need to be trained again in subsequent tests. The test time of 420 sets of test data in this experiment is about 0.013s (see the classRF_predict in Fig 23 for details), therefore, it takes about 0.03ms to test one fault data. In terms of channel transmission, the current channel delay is below 20 ms [27]. In summary, the protection time of the algorithm proposed in this paper is about 23ms, and the current computer hardware conditions can meet the fast-moving requirements.

#### 6.4.4 Comparison with other HVDC transmission line intelligent fault identification algorithms

Compared to published work on intelligent protection schemes for HVDC systems [18–20], the methods presented herein shows higher capabilities with fewer measurements. The scheme of [18] can accurately identify whether the fault is located in the area, outside the rectifier side area or outside the inverter side area, but the fault selection is an important fault feature and is not recognized. In addition to DC voltage and current, SVM-based solutions [19] also require AC RMS voltage. It is worth noting that, unlike the solution proposed in this paper, the scheme of [19] has not been verified in distinguishing between internal faults and external faults, and it seems that this capability is not designed for the scheme. In addition, transition resistance is one of the important features of HVDC fault identification, the schemes of [18,19] also do not evaluate the influence of different fault resistance values on the proposed algorithm, and their noise interference capability needs to be verified.

Document [20] uses K-means data description (KMDD) method to detect and classify internal faults in bipolar HVDC transmission lines, and has outstanding capabilities in real-time detection and anti-noise interference. However, the anti-transitional resistance capability of this method needs to be improved, and it only discusses the identification of internal faults, the capability to identify internal and external faults has not been verified.

At the same time, in order to verify the identification effect of the model in this paper, the PNN, ELM, BP and RBF networks are selected to diagnose and identify the HVDC transmission line faults discussed in this paper, and the test results are shown in Table 10. It can be known from Table 10 that the identification rate of the HVDC transmission line fault identification model based on random forest is the highest among the five models. It shows that the model of the method proposed in this paper has better identification rate and superior performance, and can effectively solve the fault identification problem of HVDC transmission lines.

**Table 10:** Comparison of recognition results.

Name	Training set recognition rate	Test set recognition rate
RF(Random Forest)	100%	100%
PNN(Probabilistic Neural Network)	100%	93.5174%
ELM(Extreme Learning Machine)	93.5976%	85.4762%
BP(Back Propagation Network)	83.2317%	85.7134%
RBF(Radical Basis Function Network)	99.1%	91.667%

## 7 Conclusion

Because the traditional traveling wave protection only uses traveling wave head information, when the amplitude of the wave head is small or the sampling data is lost, the reliability of the protection algorithm is insufficient. In order to remedy the current problem of having been buffeted by competing requirements for both protection sensitivity and quick reaction of HVDC transmission lines simultaneously, a new principle of intelligent fault identification for HVDC transmission lines based on multi-scale S-transform fluctuation index, energy sum ratio and random forest is proposed in this paper by using the ability of intelligent algorithms to learn features. The new principle finally realizes the internal and external fault identification and fault pole selection of HDVC transmission lines by extracting the fault traveling wave signals at different scales by S transform, calculating the fluctuation index, energy sum ratio of the sampled data within 2 ms after the fault and constructing a fault feature sample set, which is trained and tested the established random forest intelligent fault identification model. Theoretical derivation and simulation experiments show that:

(1) In this paper, the fluctuation index is used to reflect the fault characteristics in the region and outside, and the energy sum ratio is used to reflect the fault pole characteristics. The multi-scale fluctuation index and the energy sum ratio are combined to form the combined feature sample set which is used as the input of the random forest intelligent model. The using of an intelligent network can simultaneously realize internal and external fault identification and fault pole selection, and can meet the fast-moving requirements. At the same time, the method does not require threshold setting, and overcomes the shortcomings of traditional protections which requires different criteria to achieve fault identification and fault poles selection, and is difficult to set the thresholds.

(2) In this paper, the multi-scale S-transform characteristic frequency information is used to improve the fault-tolerance of the protection algorithm, and the random forest learning and generalization abilities are used to realize the intelligent identification of HVDC transmission line faults, which overcomes the problems of low reliability and poor fault tolerance of traditional traveling wave protection which only uses traveling wave head information, and it has a strong anti-noise ability. A large number of simulations show that the proposed algorithm is insusceptible to data loss, transitional resistances, noise interference and other factors. It can accurately and quickly identify the internal or external faults while achieving fault pole selection.

## Supporting information

S1 Data (ZIP)
