# Supplementary material for: A novel intelligent fault identification method based on random forests for HVDC transmission lines
Source: PLoS One. 2020 Mar 26;15(3):e0230717. doi: 10.1371/journal.pone.0230717 (PMC7098650; doi:10.1371/journal.pone.0230717)

**
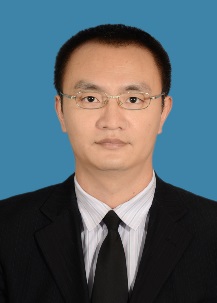
**HAO WU (Non-member) He received the Ph.D. degrees in the automation of electric power systems from Southwest Jiaotong University, Chengdu, China, in 2016, and is presently an Associate Professor with the School of Automation and Information Engineering, Sichuan University of Science & Engineering, Zigong, China. His research areas include power system protection and control, power systems simulation and modeling. Email: [wuhao801212@163.com](mailto:wuhao801212@163.com)

**QIAOMEI WANG**
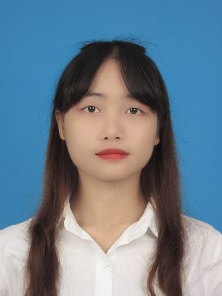
 (Non-member) She received the B.Sc. degree in electrical engineering and automation from Sichuan University of Science and Engineering, China, in 2018 and is presently pursuing the M.Sc. degree in the intelligent control of electric power systems at Sichuan University of Science and Engineering, Zigong, China. Her research interests include power system protection and smart substations. Email: wqm478712791@163.com


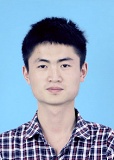
**KUNJIAN YU** (Non-member) He received a B.Sc. degree in electrical engineering and automation from Chengdu University, Chengdu, China, in 2017 and is currently pursuing an M.Sc. degree in the intelligent control of electric power systems at Sichuan University of Science and Engineering, Zigong, China. His research interests include smart substations and image processing. Email: 18582457396@163.com


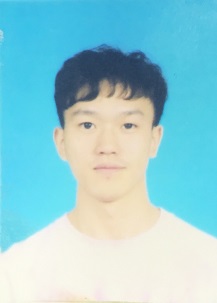
**XIAOTAO HU** (Non-member) He received a B.Sc. degree in electrical engineering and automation from Binzhou University, Binzhou, China, in 2016 and is currently pursuing an M.Sc. degree in the intelligent control of electric power systems at Sichuan University of Science and Engineering, Zigong, China. His research interests include pattern recognition and smart substations. Email: ray_scqhg@163.com

**MAOXIA RAN** (Non-member) She received the B.Sc. degree in electrical engineering and automation from Sichuan University of Science and Engineering, China, in 2018 and is presently pursuing the M.Sc. degree in control engineering at Sichuan University of Science and Engineering, Zigong, China. Her research interests is signal processing. Email: ranmaoxia112@163.com com
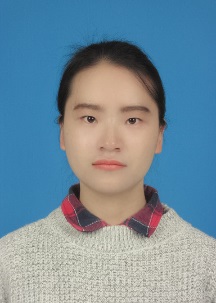

Supplement: S1 Data — (ZIP) [file pone.0230717.s001.zip › Supporting information/Author Information.docx]
