# Supplementary material for: A novel intelligent fault identification method based on random forests for HVDC transmission lines
Source: PLoS One. 2020 Mar 26;15(3):e0230717. doi: 10.1371/journal.pone.0230717 (PMC7098650; doi:10.1371/journal.pone.0230717)
Supplement: S1 Data — (ZIP) [file pone.0230717.s001.zip › Supporting information/PONE-D-19-24372- Financial Disclosure.docx]

**Funding Statement**

This research was supported by National Natural Science Foundation of China (Grant Nos. 11705122), the artificial intelligence key laboratory of Sichuan province Foundation (2017RYY02), and the Project of Sichuan provincial science and Technology Department (Grant No. 2017JY0338，2019YJ0477，2018GZDZX0043)，Enterprise informatization and Internet of things measurement and control technology key laboratory project of Sichuan provincial university (2018WZY01)，Sichuan University of Science and Engineering talent introduction project (2017RCL53) and the Project of Sichuan Provincial Academician (Expert) workstation of Sichuan University of Science and Engineering(2018YSGZZ04).
