# Supplementary figures and images for: A novel intelligent fault identification method based on random forests for HVDC transmission lines
Source: PLoS One. 2020 Mar 26;15(3):e0230717. doi: 10.1371/journal.pone.0230717 (PMC7098650; doi:10.1371/journal.pone.0230717)

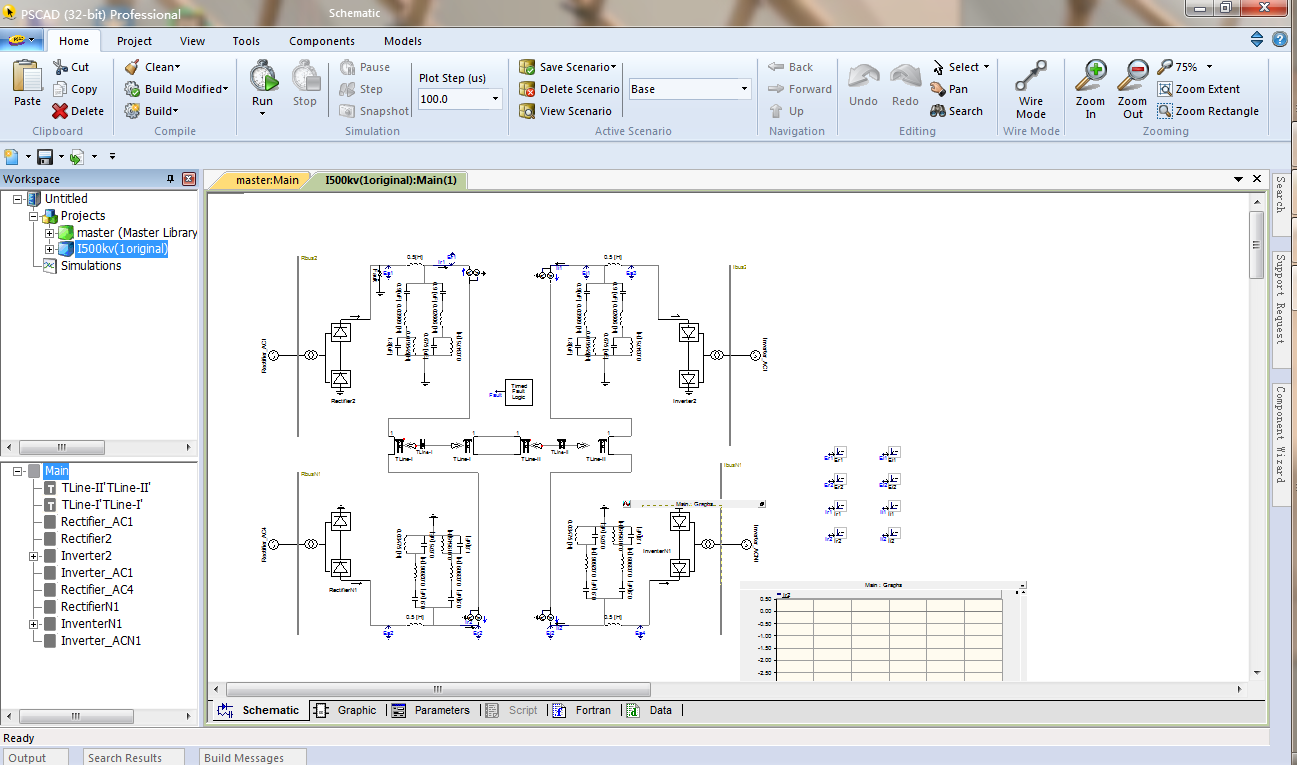

Supplement: S1 Data — (ZIP) [file pone.0230717.s001.zip › Supporting information/model.tif]
